# Skin Aging, Cellular Senescence and Natural Polyphenols

**DOI:** 10.3390/ijms222312641

**Published:** 2021-11-23

**Authors:** Erika Csekes, Lucia Račková

**Affiliations:** Centre of Experimental Medicine, Institute of Experimental Pharmacology and Toxicology, Slovak Academy of Sciences, Dúbravská Cesta 9, 841 04 Bratislava, Slovakia

**Keywords:** skin aging, senescence, anti-senescence, natural polyphenols

## Abstract

The skin, being the barrier organ of the body, is constitutively exposed to various stimuli impacting its morphology and function. Senescent cells have been found to accumulate with age and may contribute to age-related skin changes and pathologies. Natural polyphenols exert many health benefits, including ameliorative effects on skin aging. By affecting molecular pathways of senescence, polyphenols are able to prevent or delay the senescence formation and, consequently, avoid or ameliorate aging and age-associated pathologies of the skin. This review aims to provide an overview of the current state of knowledge in skin aging and cellular senescence, and to summarize the recent in vitro studies related to the anti-senescent mechanisms of natural polyphenols carried out on keratinocytes, melanocytes and fibroblasts. Aged skin in the context of the COVID-19 pandemic will be also discussed.

## 1. Introduction

Aging is a process defined as the time-dependent persistent change in functionality and reproducibility (of all higher organisms) related to an increased probability of morbidity and mortality [1]. The human skin is constantly exposed to internal and external stimuli that have an impact on its functionality with the progression of the age, manifesting as wrinkling, dry skin, a reduced barrier integrity and thinning of the epidermis.

On the cellular level, aging was first described by Hayflick and Moorhead [2], who demonstrated that human primary fibroblasts have a limited ability to divide. This is known as the Hayflick limit and originates from the inability of telomeres to maintain their lengths due to the replication process. Consequently, cells lose their proliferative capacity and enter a state of irreversible cell cycle arrest, later termed cellular or replicative senescence [1,3,4].

Senescent cells are characterized by their inability to proliferate, resistance to apoptosis and secretion of factors that promote inflammation and tissue deterioration [1,5,6]. It has been shown that senescent cells accumulate with age and may contribute to age-related skin changes and pathologies. However, whether senescent cells are the main cause of aging or whether they are the result of aging and only contribute to aging deterioration and the development of age-related diseases is still under investigation.

Natural compounds have been used in dermatology as oral dietary supplements or topical formulations for a long time. Polyphenols are the most abundant natural biochemicals found in fruits, vegetables seeds and spices, as well as red wine, coffee and cocoa. Many beneficial effects of polyphenols have been shown, including antioxidant and free radical scavenging activity, anti-tumor and anti-inflammatory properties and anti-thrombotic and anti-microbial activity [7,8,9]. Moreover, there is growing evidence that polyphenols can slow down or prevent the aging-related deterioration of the appearance and function of the skin [10].

In this review, we aim to provide an overview of the current state of knowledge in skin aging, and hallmarks of senescent skin cells will be discussed. We also focus on the mechanisms regarding how polyphenols operate cellular senescence within the most studied cell types of the skin—keratinocytes, melanocytes and fibroblasts. Moreover, since aged skin is more vulnerable to infection by viruses, aged skin in the context of the COVID-19 pandemic will be also discussed.

## 2. Cellular Senescence in Skin Aging

The process of aging itself involves different interdependent hallmarks on a molecular, cellular and organ level. On a cellular level, the aging of mitotic cells is defined as cellular senescence (or cell aging), which is also thought to contribute to organismic aging. It represents a complex process of permanent cells cycle arrest, while the cells remain metabolically active. It is also used as a simple experimental model of aging human tissues. Under in vitro conditions, several types of cellular stressors can trigger senescence [1,5,11]; however, the identification of unique senescence markers, particularly in vivo, is still under investigation. Under physiological conditions, the signal of senescence induction is telomere shortening and dysfunction (so called replicative senescence) [12,13,14]. However, senescence can also be induced prematurely as a consequence of direct and persistent DNA damage [15], oxidative stress [16], strong mitogenic signal, the inactivation of a tumor suppressor (such as phosphatase and tensin homolog (PTEN)) and/or oncogenes (such as Raf or BRAF) (so called oncogene-induced senescence, OIS) [15,17], mitochondrial dysfunction (named as senescence associated with mitochondrial dysfunction, MiDAS) [18], epigenetically (induced by inhibitors of DNA methylases or histone deacetylases) [19], by a primary senescent cell producing a senescence-associated secretory phenotype (SASP, so called paracrine senescence) [20] and by chemotherapy (therapy-induced senescence, TIS) [19]. Senescence has several beneficial functions for the organism (for instance, it acts against tumorigenesis due to irreversible proliferation arrest); however, there is increasing evidence suggesting that senescent cells accumulate in aging tissues and organs, thereby impairing physiological processes, including regeneration, and contributing to organismal aging [1,21,22].

Skin aging, like aging of the other organs, is characterized by a progressive loss of functionality and regenerative potential. It is a multi-factorial process that affects nearly every aspect of its biology and function. The skin, our mechanically protective and flexible barrier organ, is the most visible organ, where all changes, including aging, are very noticeable. The aging process of skin can be described as intrinsic and extrinsic. Intrinsic or chronological skin aging is an inevitable process of chronological and physiological alterations. Intrinsic factors that drive skin aging are time, genetic factors and hormones. It is also an oxidative process that is related to a progressive, age-related decline in antioxidant capacity and an increased production of reactive oxygen species (ROS) [23]. The clinical signs corresponding to intrinsic skin aging are fine lines, xerosis (dry skin) and laxity [24]. However, extrinsic aging is restricted mostly to exposed sites, such as the face, neck and hands, and is predominantly manifested as coarse wrinkles, irregular pigmentation and lentigines (or age spots). The exposome contributing to extrinsic skin aging involves sunlight, air pollution, cigarette smoke, nutritional factors, temperature, stress and lack of sleep [25]. Thus, extrinsic aging is thought to be laid over the intrinsic one and depends on the intensity and duration of exposure to environmental factors and on the skin type. Pollution and cigarette smoke are well-known external factors that accelerate skin aging; however, the most significant extrinsic aging factor is still UV radiation (known as photoaging), which causes DNA damage and oxidative damage, inducing cellular senescence [26,27].

The human skin represents a dynamic and complex organ with a unique structure. It consists of different cell types and compartments with different functions. The outermost layer, the epidermis, consists of four sublayers—namely, stratum corneum, stratum granulosum, stratum spinosum and stratum basalis—and four major cell types—keratinocytes, melanocytes, Langerhans cells and Merkel cells. The border between the epidermis and dermis, the epidermal–dermal junction, represents an aggregation of proteins and structures known as the basement membrane. Below the basement membrane, there is the underlying dermis, which provides structural support, as well as nutrition and circulation in the skin [28]. The dermis comprises, besides dendritic cells, macrophages and mast cells, primarily from fibroblasts, which produce an interconnected extracellular matrix of collagenous and elastic fibers. The dermis also contains blood and lymph vessels, nerves, hair follicles and sweat glands.

Aging appears to affect all skin layers, and is manifested as alterations in terms of their structure and function [29]. The aged epidermis shows a lessened capacity for barrier function and restoration following insult [30]. A lipid processing decline, as well as a decrease in the epidermal levels of CD44 glycoprotein, a regulator of keratinocytes proliferation, and the maintenance of local hyaluronic acid homeostasis, have been shown to contribute to this decline [31,32]. In addition, it has been shown that, with age, the proliferation of cells in the basal layer declines. The epidermis then decreases in thickness, and the contact surface area between the dermis and epidermis diminishes, resulting in a smaller exchange surface for nutrition supply to the epidermis and a further weakened ability of basal cell proliferation [33,34]. Besides the epidermis, both the epidermal–dermal junction and dermis also become thinner. The flattening of the epidermal–dermal junction leads to fewer cells, less nutrition and less oxygen, resulting in wrinkle formation. The dermal extracellular matrix (ECM) also exhibits structural and functional changes in both intrinsically and extrinsically aged skin. These include an altered accumulation of type I and type III collagens and changes in the ratio of type I/III [35], an impaired synthesis of these ECM molecules [36] and changes in the elastic fiber organization [37,38]. The decrease in the number of fibroblasts also contributes to alterations and the degradation of ECM, which manifests as progressed dermal thinning, increased wrinkling and a loss of elasticity [26].

Besides being an aesthetic issue with a related psychological and social impact, skin aging also increases the risk of susceptibility to infections, chronic wounds, such as venous, pressure or diabetic foot ulcers, and various types of dermatitis and malignancies, including melanoma [29,39].

Increasing evidence suggests that senescent cells accumulate in chronologically aged skin, as well as prematurely aged skin, and may contribute to age-related skin changes and pathologies. The accumulating senescent keratinocytes and fibroblasts in skin produce cytokines, extracellular matrix-modifying enzymes and other molecules that can act at a distance, and can thus exert long-ranging effects on the microenvironment of neighboring cells [13,21,40,41,42,43]. Both intrinsic and extrinsic factors can induce permanent senescence in skin cells, resulting from the shortening of telomeres, mitochondrial impairment and upregulation of DNA damage response signaling, finally leading to the cell cycle arrest [44,45]. Consequently, the presence of senescent keratinocytes and fibroblasts has been suggested to contribute to the decline in the integrity and function of the skin [46]. Moreover, melanocytes were also found to both display the markers of senescence, including an elevated p16^INK4A^, reduced high-mobility group box 1 (HMGB1) and dysfunctional telomeres, and to affect the basal keratinocyte proliferation via the activation of CXC chemokine receptor 3-dependent mitochondrial ROS, thus contributing to epidermal atrophy [47].

Moreover, the proliferative arrest of cultured skin cells due to replicative or stress-induced senescence also represents a useful model for the study of aging-related processes in the skin [48]. Accordingly, it has been shown, using in vitro models, that UVB-exposed skin cell types (fibroblasts, keratinocytes) exhibit DNA damage and cell cycle arrest and express senescence biomarkers, such as increased senescence-associated β-galactosidase (SA-β-Gal) activity, p16^INK4A^, p21^Waf-1^, p53 activation and lamin B1 downregulation [49,50,51]. In vivo, a chronic low dose exposure to UVB resulted in the accumulation of DNA damage and loss of lamin B1 corresponding to senescent cells within the mouse epidermis, but not the dermis [51]. Low doses of UVB irradiation also induce stress-induced premature senescence (SIPS) in keratinocytes. This is observed via an increase in SA-β-Gal activity and a sustained increase in p21^Waf-1^ and p53 expression, and is insulin-like growth factor-1 receptor (IGF-1R)-dependent [50]. UVB-SIPS has also been described in melanocytes, as shown by the overexpression of p53 and p21^Waf-1^, associated with the hypo-phosphorylation of pRb [52].

### 2.1. Biomarkers of Cellular Senescence in the Skin

Senescent cells, besides being characterized by permanent proliferation arrest, confess functional and morphological changes. Many of these changes are used as markers of senescence; however, a universal senescence biomarker is still lacking.

#### 2.1.1. Senescence-Associated Ultrastructural Changes

Senescence in the skin cells is accompanied by the following ultrastructural changes:

- **Hypertrophy and increased granularity.** In senescent cells, both an increase in size and, if adherent cells, a flattening of the shape can be observed [3]. For these changes, the activation of the mammalian target of the rapamycin (mTOR) signaling pathway is responsible [53]. These morphological changes are easily detectable by light microscopy and quantified by flow cytometry (as an increase in forward scatter, FSC parameters). However, though in situ and in vivo quantification can be a problem, changes in plasma membrane protein expression represent a promising new biomarker of senescence [54]. The size increase by up to nine times was found in senescent fibroblasts [3]. The size increasing with other senescence markers was also confirmed in aged keratinocytes [55] and in a model of UVB-induced senescence in human melanocytes [56]. The senescent nuclei of skin cells also showed hypertrophy. In particular, the mean nuclear area of fibroblasts was shown to be 255 μm^2^ at early passage, compared to 293 μm^2^ at later passage [57]. The increase in granular content in senescent cells can be monitored by transmission electron microscopy as intracellular electron-dense particles [58]. However, cell granularity levels can also be conveniently detected by flow cytometry as an increase in the side scatter (SSC) parameter. The increase in granularity in senescent human fibroblasts is a result of the intracellular deposit formation, including lipofuscin in lysosomes and glycogen particles [59,60]. In a model of the UVB-promoted senescence of melanocytes, the cell population showing a high granularity was mostly growth-arrested at G2/M phase [61];
- **Increase in lysosomal mass and SA-β-Gal staining.** The increase in the lysosomal mass in senescent cells is associated with the accumulation of old lysosomes and increased lysosomal biogenesis. Accumulated lipofuscin may be in line with the impaired lysosomal turnover mechanism [62]. Lysosomal biogenesis is largely controlled by the transcription factor EB (TFEB), an effector protein within the mTOR signaling pathway that regulates multiple lysosomal proteins. During senescence, it tends to be up- or down-regulated, making it difficult to use as a marker of senescence [63,64]. Alternatively, the detection of **lipofuscin** content can be used as a biomarker of lysosome accumulation, either by its typical autofluorescence properties and fluorescence-based methods, or by selective staining with Sudan black B, allowing for detection in cells, tissues and body fluids [65]. The term lipofuscin originates from the Greek words “lipo” (fat) and “fuscus” (dark) [1]. In addition, it is referred to as “aging fluorophore” or “aging pigment”. It is an insoluble material that mainly consists of a highly oxidized and crosslinked substrate, which are proteins, lipids and sugars. Transition metals also bind to lipofuscin and increase its intracellular cytotoxicity through the catalysis of ROS formation by the Fenton reaction. Lipofuscin is also present in small amounts in the cytosol (about 1% of the total intracellular content), while its cytotoxicity is suppressed by the macroautophagy activity of the cell. It preferentially accumulates in postmitotic tissue cells, such as neurons or muscle cells, which do not divide and are therefore unable to dilute the products of their damage (in the sense of the so called “garbage catastrophe theory of aging”) [66]. However, lipofuscin has also been shown to accumulate during the replicative senescence of human fibroblasts [67]. Lipofuscin accumulation has been detected in the basal layers of the aged epidermis [68]. The phototoxicity of visible light has been linked to accumulated lipofuscin in skin cells due to oxidative damage in nucleic acids, lipids and proteins, generating premutagenic DNA lesions and releasing pro-inflammatory cytokines and metalloproteinases, consequently exaggerating cell damage and skin aging [69]. The increase in the size and shape of lysosomes is mostly associated with an increase in the activity of the lysosomal enzyme, **senescence-associated b-galactosidase** (SA-β-Gal). Since SA-β-Gal is upregulated in senescent cells, its residual activity can be monitored at suboptimal pH 6.0. It is the most widely used marker of senescence in culture and tissue samples [70]. However, factors such as confluence during cell culture may contribute to the detection of a false positive signal [71]. Furthermore, this assay requires active enzymatic SA-β-Gal activity, which is often lost in fixed or cryopreserved tissues [72,73]. In addition, non-specific SA-β-Gal activity was detected in the early passage of adult melanocytes proliferating in culture [21]. The available methodologies allow, depending on the properties of a specific synthetic b-galactosidase substrate, for the quantification of senescent cells in in vitro models or tissues of an aged organism using a combination of flow cytometry or spectrofluorimetry with high-content image analysis [74]. SA-β-Gal activity has been successfully confirmed in human fibroblasts and keratinocytes undergoing replicative senescence in vitro, in skin samples or in the cells isolated from aged individuals [75,76,77,78,79]. Furthermore, SA-β-Gal has been used to confirm premature senescence in cultured fibroblasts, keratinocytes and melanocytes cells in response to various stressors, including UV light [43,50,80,81,82,83], cigarette smoke [84,85], ionization radiation [86], oxidants [87,88,89] or anticancer drugs [90];
- **Accumulation of mitochondria.** Senescent cells usually have a higher number of mitochondria and also display organelle enlargement [91]. Highly elongated or enlarged giant mitochondria were observed in senescent human foreskin diploid fibroblasts, with their population doubling between 90 and 94 times [91]. However, the mitochondrial membrane potential is reduced, which is associated with an increased ROS production and the release of mitochondrial enzymes, such as endonuclease G [92,93]. This is mainly due to the reduced specific autophagy of mitochondria, mitophagy, causing old and dysfunctional mitochondria to accumulate [94]. Reduced mitochondrial scission and excessive fusion, which likely occur to compensate for the dysfunction of mitochondria in senescent cells and to protect them from apoptosis and mitophagy [95], contribute to mitochondria enlargement [96]. Dysfunctional mitochondria also represent a major source of elevated ROS production in senescent cells, another important hallmark of senescence [97]. There is also a strong link between ROS-related mitochondrial damage and photoaging. The repetitive UVA exposure was found to be accompanied by a rise in mitochondrial DNA mutations. In particular, the photoaged skin comprises up to 10-fold more frequent mitochondrial DNA mutations compared to sun-protected skin [98,99,100]. Moreover, mitochondrial DNA mutations are positively associated with matrix metalloproteinase-1 (MMP-1) levels without the related increase in MMP-1 specific tissue inhibitors [101];
- **Nuclear changes.** Senescent nuclei may contain so termed senescence-associated heterochromatin foci (SAHFs), the silent domains that co-localize with H3K9me3 and heterochromatin protein 1 (HP1) and may lock cells in a senescent state by transcriptionally repressing genes involved in cell proliferation [102]. The SAHFs can be visualized by staining with 4′, 6-diamidino-2-phenylindole (DAPI) and appear as fluorescent spots representing condensed chromatin domains that block certain genes required for proliferation [12]. The long-term monitoring of senescent cells in vitro revealed the progressive proteolysis of histones 3 and 4 without DNA loss. A reduced histone content was also observed in nevus melanocytes, as compared to neighboring non-senescent melanocytes and keratinocytes in vivo [103]. These studies confirm the dramatic structural changes in chromatin in senescent cells.
  SAHFs are also implicated in the downregulation of lamin B1, a structural protein of the nuclear lamina/membrane [22,104]. Lamin B1 has been shown to be downregulated in cells undergoing mainly replicative senescence and OIS and UV-induced senescence in vitro [26,103,105,106], and also decline during the chronological aging of human skin in vivo [105], in senescent melanocytes within human nevi [103] and in the UV-exposed mouse skin epidermis [51]. The destabilization of nuclear integrity leads to other changes, such as a loss of constitutive heterochromatin condensation and the formation of cytoplasmic chromatin fragments that contain epigenetic tags associated with DNA damage [103]. SAHFs production is thought to be a compensatory mechanism that maintains constitutive heterochromatin [107]. SAHFs, however, are not a universal marker of senescence, but are observed especially in the case of OIS [17]. Lamin B1 downregulation preferentially depends on p53 and p16, but is independent of other signaling pathways associated with senescence, such as p38 mitogen-activated protein kinases (MAPK), NF-κB and DNA damage response (DDR) [108].

#### 2.1.2. Changes in Cyclin-Dependent Kinase Inhibitors (CDKIs) Expression

CDKs phosphorylate and regulate several proteins involved in cell cycle progression. The major CDKIs responsible for cell cycle arrest during senescence are encoded in the loci *CDKN2A* (p16^INK4A^), *CDKN2B* (p15^INK4b^) and *CDKN1A* (p21^CIP/Waf-1^) [22].

**p16^INK4A^** directly interacts with and inhibits CDK4/6. It is considered to be the unique and specific marker of senescence and is also widely used to detect senescence in vivo [109,110,111]. Experimental evidence suggests that epigenetic changes are the major triggers of p16^INK4A^ upregulation, but other regulatory factors, ranging from promoter accessibility to protein stability, have been also reported [22,112,113,114].

**p21^CIP/Waf-1^** is an inhibitor of several cyclin-dependent kinases, but, unexpectedly, is also required for cell cycle progression [115]. Although it is upregulated by a variety of senescence-inducing signals, it is part of the more general DDR and is regulated by the direct transactivation of **p53**, making it less useful as a specific marker of senescence. p21**^CIP/Waf-1^** can also be activated by a mechanism independent of p53, via TNF-β and Sp1 [115,116,117].

**Figure 1:**
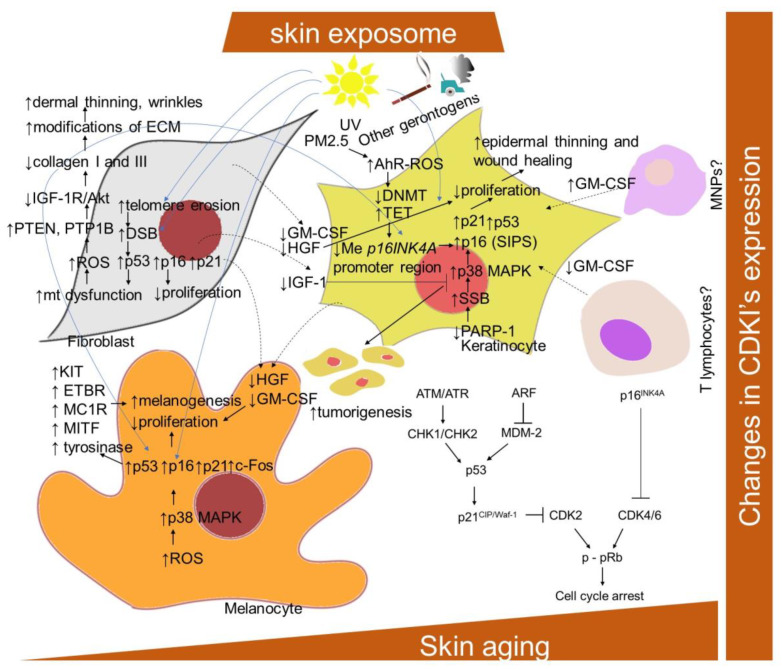
Schematic representation of aging- and senescence-related changes associated with CDKIs expression. AhR-ROS, aryl hydrocarbon receptor and ROS-mediated pathway; Akt, protein kinase B; ATM, protein kinase ataxia-telangiectasia mutated; ATR, ATM and Rad3-related protein kinase; ARF, alternative reading frame protein; CDK, cyclin-dependent kinase; c-Fos, proto-oncogene; CDKI, cyclin-dependent kinase inhibitor; DSB, DNA double-strand break; DNMT, DNA methyltransferase; ECM, extracellular matrix; ETBR, endothelin–endothelin receptor B; GM-CSF, granulocyte–macrophage colony-stimulating factor; HGF, hepatocyte growth factor; IGF-1R, insulin-like growth factor-1 receptor; mt, mitochondria; KIT, transmembrane protein with tyrosine kinase activity; MAPK, mitogen-activated protein kinase; MDM-2; mouse double minute 2 homolog; MITF, microphthalmia-associated transcription factor; MNPs, mononuclear phagocytes; NRAS and BRAF, proto-oncogenes; PARP-1, poly-(ADP-ribose) polymerase 1; PTEN, phosphatase and tensin homolog; p-pRb, phosphorylated retinoblastoma protein; PTP1B, protein tyrosine phosphatase 1B; ROS, reactive oxygen species; PM2.5, particular matter 2.5; SSB, DNA single-strand break; TET, ten–eleven translocation enzyme.

CDKIs play a critical role in two main tumor suppressor pathways that regulate the proliferative arrest during senescence: p53/p21^CIP/Waf-1^ and p16^INK4A^/**pRb** [104,118,119] (Figure 1). Both pathways can be activated in parallel and can also induce cell cycle arrest independently of each other. They represent complex pathways with regulators and effector molecules that intersect with each other and control the development of senescence by causing changes in gene expression. p53 and retinoblastoma protein, pRb, are the major transcriptional regulators. p21^CIP/Waf-1^ is called a downstream effector p53, whereas p16^INK4A^ primarily functions in cell cycle control as a negative regulator of the prominent pRb/E2F pathway. The signaling process of these paths is as follows: In the p53/p21^CIP/Waf-1^ signaling pathway, p53 is regulated by DDR signaling pathways, as well as the ARF (alternative reading frame) pathway [20]. The DDR pathway is mediated by ataxia-telangiectasia mutated and Rad3-related (ATM/ATR) kinase and checkpoint protein 1/checkpoint protein 2 (CHK1/CHK2) kinase, which stabilize p53 by phosphorylation. The ARF pathway activates p53 by inhibiting Mdm-2, a ubiquitin ligase that facilitates p53 degradation. When p53 is stabilized, the cell cycle inhibitor p21^CIP/Waf-1^ is activated. In particular, p21^CIP/Waf-1^ inactivates pRb through the inactivation of the cyclin/CDK complex, which is responsible for the phosphorylation and activation of pRb, thereby disabling pRb via p53 and preventing DNA synthesis by the pRb-activated E2F factors [22,104]. However, pRb can be inactivated by p21^CIP/Waf-1^ or p16^INK4A^. In both, their cell cycle inhibitory effect is mediated by the inhibition of the CDK/pRB/E2F pathway [12]. PTEN/p27^Kip1^ is another regulatory pathway; however, its exact function is not completely understood [120].

It has been found that, though keratinocytes and fibroblasts express the same senescent markers, they do not share the same pathway of DNA damage [121]. The senescence in fibroblasts is established following the telomeric-deprotection-induced generation of double-strand breaks. However, senescent keratinocytes accumulate single-strand breaks following failure in repair action initiated by poly(ADP)ribose polymerase (PARP), predominantly PARP1, leading to the p38 mitogen-activated protein kinases (MAPK) activation and upregulation of p16^INK4A^. In addition, exposure to PM2.5 was shown to upregulate p16^INK4A^ in keratinocytes epigenetically through the aryl hydrocarbon receptor (AhR)/ROS-mediated downregulation of DNA methyltransferase (DNMT) expression and an increase in DNA demethylase (ten–eleven translocation; TET) expression, leading to a hypomethylation of the *p16^INK4A^* promoter region [122].

The expression levels of p16^INK4A^ have shown efficiency as a robust marker of both in vitro and in vivo skin cellular aging, as well as in skin equivalent models [40,123,124,125]. p16^INK4A^-positive cells also accumulate in precancerous lesions, including melanocyte-rich benign human nevi, caused by activating mutations in NRAS or BRAF [103,126]. Considering the role of p16^INK4A^, being deeply involved in the senescence mechanism, it is not surprising that this locus is frequently mutated in a variety of human cancers, including skin epithelial tumors [127,128].

Extrinsic stressors, such as UV and ionization radiation, also simultaneously upregulated p16^INK4A^, p21^CIP/Waf-1^ and p53 in human fibroblasts, keratinocytes and prematurely aged skin [26,83,129,130,131]. Nevertheless, the dependence on the p16^INK4A^ family of tumor suppressor proteins activated upstream to pRb has been suggested to distinguish stress-induced premature senescence (SIPS) from replicative senescence [132]. A significant upregulation of p16^INK4A^ was also observed in H_2_O_2_-treated melanocytes, as well as in equally treated human keratinocytes and fibroblasts [133]. The knockdown of p16^INK4A^ caused an elevation of intracellular ROS and oxidative DNA damage (measured as 8-oxoguanine) in diverse skin cells and whole skin, which was further boosted by H_2_O_2_ treatment. Interestingly, melanocytes showed an increased susceptibility to p16^INK4A^-depletion-dependent oxidative damage, which might explain why the impaired expression of p16^INK4A^ predisposes to melanoma over other cancers. Consistently, p16^INK4A^-positive epidermal cells, identified as mostly melanocytes, were also significantly correlated with enhanced facial wrinkling and a higher perceived age in the analysis of sun-protected upper-inner arm skin biopsies from 178 participants (aged 45–81 years) [134]. In addition, p16^INK4A^-positive epidermal and dermal cells were significantly associated with age-related elastic fiber morphologic features; in particular, longer and a greater number of elastic fibers. Moreover, Victorelli and colleagues [47] showed that melanocytes are the only epidermal cell type to express the senescence marker p16^INK4A^ during human skin aging and can thus drive the skin aging process. Nevertheless, in contrast to other senescent cell types, and due to the effect of UVB irradiation, the senescent melanocytes have reduced or absent levels of the CDKIs p27^Kip1^ and p21^CIP/Waf-1^ [135]. In addition, in melanocytes, a link between p53 and increased melanogenesis in senescent cells has been provided [61]. In particular, the UVB irradiation of melanocytes was shown to upregulate p53, p21^Waf-1^ and c-Fos, and to inhibit retinoblastoma phosphorylation. Accordingly, a repeated exposure of human melanocytes to UVB leads to melanocytes senescence and an increased p53 expression-mediated pigmentation. In addition, a decrease in epidermal proliferation and differentiation accompanied by an enhanced accumulation of senescence markers, including p16^INK4A^, during aging might be essentially influenced by a decrease in the production of IGF-1 by dermal fibroblasts suppressing collagen synthesis [136] (Figure 1). This was explained by enhanced mitochondrial superoxide production activating phosphatases protein tyrosine phosphatase 1B (PTP1B) and PTEN leading to a lessening of IGF-1R/Akt signaling. In addition, a link between the reduced production of IGF-1 by senescent fibroblasts in the dermis of geriatric skin and an increased risk of skin tumorigenesis has been proposed [137]. This can be explained by evidence that keratinocytes with inactive IGF-1 receptors show partial defects in nucleotide excision repair and DNA damage checkpoint signaling.

Furthermore, the production of other mitogens, such as hepatocyte growth factor (HGF) and granulocyte–macrophage colony-stimulating factor (GM-CSF), was lowered in reconstructed human skin containing fibroblasts from an aged donor. With regard to the suggested decrease in GM-CSF and HGF levels in the aged skin, the wound healing process [138,139] as well as melanocytes proliferation, might also be affected [140]. This is contradictory to the established increase in both mitogens within the SASP program of senescent fibroblasts [141,142]. Nevertheless, a decrease in mitogens was correlated with a reduced dermal cell number, decrease in collagen I fibrils and decreased epidermal thickness [143]. In addition, changes in mitogen levels might result from senescence-related changes in other cells secreting them, such as T lymphocytes, endothelial cells and mononuclear phagocytes.

#### 2.1.3. Changes in Apoptosis Resistance

A resistance to apoptosis is a typical characteristic of senescent cells associated with an upregulation of factors responsible for survival [4]. Such factors include Bcl-2 family proteins, ephrins, phosphoinositide 3-kinases (PI3K), p21^CIP/Waf-1^ and plasminogen-activated inhibitor-2 [144]. p21^CIP/Waf1^ protects against apoptosis by suppressing the activation of c-Jun N-terminal kinase (JNK) and caspases [145] and heat shock protein 90 (HSP90) via phosphorylated Akt (P-Akt) stabilization [146]. In addition, senescent normal human fibroblasts might fail to upregulate p53, or are preferentially recruited to the promoter of genes for cell cycle arrest (*p21^CIP/Waf1^* and *GADD45*), but not those for apoptosis regulators (*TNFRSF10b*, *TNFRSF6* and *PUMA*) [147].

**Figure 2:**
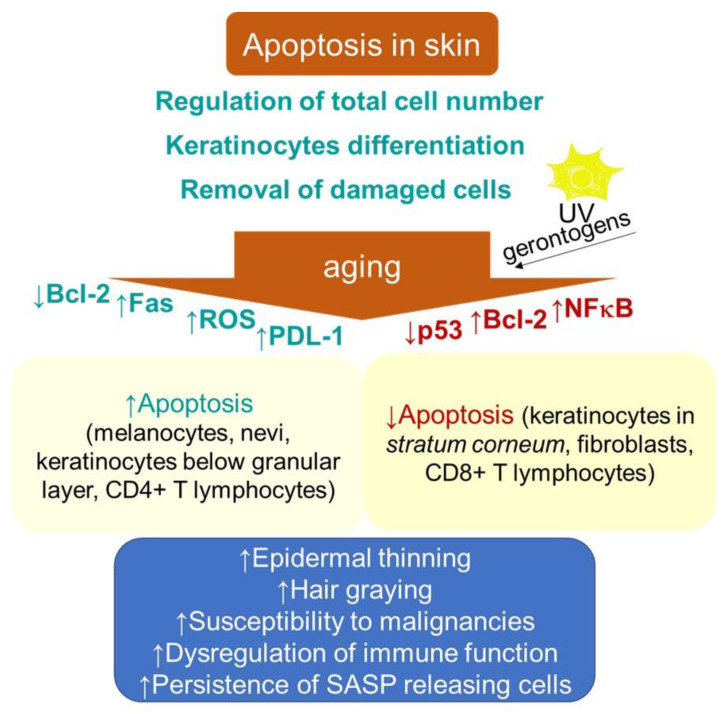
Schematic representation of aging- and senescence-related changes in skin associated with apoptosis. Bcl-2, B cell lymphoma 2; Fas, cell surface death receptor; NF-κB, nuclear factor kappa-light-chain-enhancer of activated B cells; PDL-1, programmed death-ligand 1; ROS, reactive oxygen species; SASP, senescence-associated secretory phenotype.

Apoptosis in skin is a process that is essential for normal epidermal function through providing a foundation for keratinocyte terminal differentiation, maintaining skin homeostasis by regulating the total cell number and removing the cells damaged by environmental stresses, thus preventing further damage (Figure 2). The aging-related thinning of the epidermis appears to correlate, besides the decrease in proliferation, with both an increase in apoptosis below the granular layer and epidermal Fas expression [148]. In addition, increased apoptosis due to a decline in Bcl-2 levels contributes to decreasing numbers of melanocytes and nevi with aging [149]. The age-related increase in oxidative stress can also be associated with hair graying, which is caused by the selective apoptosis of hair follicle melanocytes [150]. These findings are in contrast to the typical senescence-associated resistance to apoptotic stimuli. Nevertheless, the rate of apoptotic-like DNA fragmentation, as part of terminal differentiation, was shown to decrease in the epidermal keratinocytes with aging [151]. Furthermore, decreased epidermal and stratum corneum cell turnover with intrinsic aging has been shown [152]. In addition, the dysregulation of apoptosis through intrinsic aging processes or through random mutations has been suggested to increase the risk of the onset of cancer [153]. In this regard, some specific factors, e.g., the epidermal milieu rich in the stem cell factor receptor c-kit, can promote the resistance of the melanoma cell to apoptosis [154,155].

Moreover, a decreased proneness to apoptosis might indicate a risk of neoplasia development following extrinsic genotoxic stresses, which, typically, is UV irradiation. The epidermis of photodamaged skin is thicker than that of intrinsically aged skin, and increased numbers of atypical melanocytes and keratinocytes may be seen [156,157].

Consistently, in contrast to young keratinocytes, where UV irradiation (100–2000 J/m^2^) induced apoptotic cell death in the G1 phase, senescent cells arrested in G1 phase showed a resistance to apoptosis. Nevertheless, the activation pattern of p53 showed subtle differences that were comparable to other cell types [158], which might indicate its differential DNA binding in senescent cells [147]. The authors suggested that the development of resistance to apoptosis in senescent keratinocytes might be an important mechanism explaining an increased vulnerability of aged skin to carcinogenesis. By contrast, the exposure of keratinocytes and epidermal equivalents to IFN-γ plus phorbol ester, 12-O-tetradecanoylyphorbol-13-acetate (TPA), inducers of a non-proliferative state resembling senescence, reduced both the transcriptional activity of p53 and its total cellular levels, resulting in the suppression of UV-induced apoptosis [159]. The irreversibly growth-arrested keratinocytes also failed to activate p53 through its acetylation of lysine-382 and phosphorylation on serine-15. Hence, the pro-apoptotic function of p53 appears to be compromised in growth-arrested keratinocytes. The analogous mechanisms of apoptosis resistance development can also be observed in cultured human fibroblasts, showing relatively lower constitutive levels of p53 compared to keratinocytes [160]. In particular, Seluanov and colleagues [161] showed that, when senescent WI-38 fibroblasts were challenged with p53-dependent apoptotic stimuli, they, in contrast to young cells undergoing apoptosis, underwent necrosis instead. However, p53-independent apoptosis was only slightly reduced [161,162]. Senescence in fibroblasts induced by H_2_O_2_ also supported survival in response to pro-apoptotic stimuli, including UVB [163] and high doses of H_2_O_2_ [164]. Thus, senescent fibroblasts are apparently unable to stabilize p53 in response to DNA damage.

The upregulation of pro-survival protein Bcl-2 can also mediate the antiapoptotic effect in senescent cells. Replicatively senescent human fibroblasts displayed a resistance to apoptosis under serum withdrawal for 2 weeks that was dependent on the maintenance of unchanged levels of the Bcl-2 protein, in contrast to young and intermediate-aged cells [165]. The increased expression of anti-apoptotic proteins can also explain the resistance of senescent fibroblasts to p53-independent apoptosis induced by staurosporin [162,166]. Earlier studies suggested that environmental gerontogenic factors can affect the proteins regulating the stress response genes, including NF-κB, AP-1, β-ZIP or C/EBP and HSF, resulting in an alteration of their structure and function [167]. Consistently, Chaturvedi and colleagues [168] showed that resistance to apoptosis in keratinocytes undergoing an induction of cell cycle arrest or senescence requires the properly regulated activation of NF-κB. Furthermore, aging can significantly affect the cell survival and death signaling of the skin-resident immune cells, which belong to characteristics of immunosenescence. It has been suggested that age-related immune dysfunction may correlate with defects (either increases or decreases) in apoptosis among different T cell subpopulations [151]. A recent study using TCRδ^CreER^R26^ZsGreen^ double transgenic mice showed that, whereas aged CD4+ memory T cells were shown to exhibit pro-apoptotic gene signatures, aged CD8+ memory T cells expressed anti-apoptotic genes [169]. Consistently, an increased expression of programmed death protein 1 (PD-1) on CD4+ T cells has been shown in the skin and peripheral blood populations of these cells in older adults, which renders them more susceptible to inhibition [170]. Furthermore, no decline in the density of T cells in human skin was found with advancing age, and the frequency of epidermal CD49a+ CD8+ resident memory T cells was increased in elderly individuals regardless of the ethnicity and decline in T cell diversity and function in blood [171]. Moreover, in contrast to the dermis, the epidermis showed a significant decrease in the CD4+/CD8+ ratio by aging (*p* = 0.0349, r = −0.4736), suggesting CD8+ T cell accumulation in this layer. Thus, in view of advanced age, the T cell immunity in the skin appears to be sustained more efficiently than the circulating T cell memory. Nevertheless, the increased age-related vulnerability to some pathologies, with respect to changes in the T cell subpopulation, e.g., 20% of metastatic melanomas showing a content of CD4+ lymphocytes with specific tumor recognition [172], remains to be clarified.

#### 2.1.4. Senescence-Associated Secretory Phenotype

Chronic low-grade inflammation, termed inflammaging, manifested by elevated serum levels of inflammatory cytokines, such as IL-6, IL-8 and TNF-α, is not only limited to systemic age-related alterations but may also concern skin aging. Notably, senescent cells accumulating in the skin during aging have a primary role in driving skin inflammaging [173]. They exhibit an altered secretome, referred to as a senescence-associated secretory phenotype (SASP), which comprises proinflammatory cytokines, chemokines, proteinases and growth factors that considerably alter the skin’s microenvironment. Due to the secretion of these factors, senescence gains pleiotropic effects. Cytokine release during DDR can have both beneficial and detrimental consequences. For instance, SASP secretion results in an increased immune clearance of potentially tumorigenic skin fibroblasts [174]. In melanocytes, IGFBP7 (insulin-like growth factor-binding protein 7) secretion factor is essential for BRAF-induced senescence [175]. Furthermore, SASP is also essential for wound healing [111]. However, the deleterious effect of SASP lies in its participation in the formation of tumors, including carcinomas of human skin [176].

**Figure 3:**
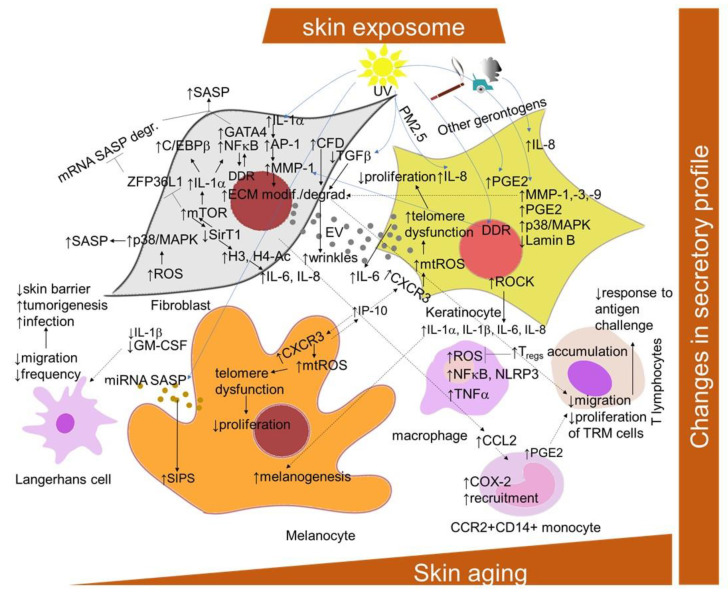
Scheme of the cellular and signaling crosstalk related to age- and senescence-related changes in SASP promotion in the skin. AP-1, activator protein-1; CXCR2 and 3, C-X-C motif chemokine receptor 2 and 3; CCL2, C-C motif chemokine ligand 2; C/EBPb, CCAAT/enhancer-binding protein beta; COX-2, cyclooxygenase 2; DDR, DNA damage response; ECM, extracellular matrix; GATA4, transcription factors GATA binding protein 4; GM-CSF, granulocyte–macrophage colony-stimulating factor; H3, histone 3; H4-Ac, acetylated histone 4; HDAC2 and 7, histone deacetylase 2 and 7; mTOR, mammalian target of rapamycin NF-κB, nuclear factor kappa-light-chain-enhancer of activated B cells; IL-6 and -8, interleukin 6 and 8; IP-10, interferon-gamma-induced protein 10; ZFP36L1, ZFP36 ring finger protein like 1; MAPK, mitogen-activated protein kinase; MMPs, matrix metalloproteinases; mtROS, mitochondrial ROS; ROCK, Rho-associated protein kinase; IGFBP7, insulin-like growth factor binding protein 7; MMPs, matrix metalloproteinases; NLRP3, NLR family pyrin domain containing 3 PGE2, prostaglandin E; PMs, particular matters; SASP, senescence-associated secretory phenotype; SirT1, silent mating type information regulation 2 homolog; SIPS, stress-induced premature senescence; TNF-α, tumor-necrosis factor alpha; TRM, tissue-resident memory T cells.

SASP is mediated primarily by the pro-inflammatory transcription factor NF-κB, which is activated in response to the DDR. Additional known regulators are the transcription factors GATA binding protein 4 (GATA4) and CCAAT/enhancer-binding protein beta (C/EBPb) [177,178] (Figure 3). The transcription of SASP genes is regulated epigenetically. The histone deacetylase SirT1 is downregulated during senescence, leading to an increased expression of the cytokines interleukin-6 (IL-6) and IL-8 through histone acetylation in the promoter regions [179]. By contrast, the specific downregulation of histone deacetylase 2 (HDAC2) or HDAC7 induced the appearance of senescence biomarkers in dermal fibroblasts [180]. mTOR kinase regulates SASP post-transcriptionally by two mechanisms: by inducing *IL-1A* translation, leading to the activation of NF-κB and C/EBPβ [177,181], or indirectly by inhibiting the RNA binding protein ZFP36 ring finger protein like 1 (ZFP36L1), which prevents SASP encoding mRNA degradation [181,182]. The studies with p38 MAPK inhibitors indicated that p38 signaling is required for the SASP in cultured fibroblasts [183]. ROS production induces the p38 MAPK pathway, which, in turn, leads to the phosphorylation and activation of other RNA binding proteins, providing stabilization of SASP-encoding mRNA [184]. Recent findings showed that Rho-associated protein kinase (ROCK) might play a role in the SASP of oral keratinocytes [185]. The pre-treatment of the cells with the ROCK inhibitor before entry into the non-proliferative state reduced the amount of IL-1α, IL-1β, IL-6 and IL-8 released by senescent cells, even in the absence of the inhibitor, without interfering with growth inhibition.

Nevertheless, the development of the SASP in skin is a result of intensive crosstalk among cellular components, including the immune cells (Figure 3). In this regard, as shown by Choi and colleagues [186], pro-inflammatory cytokine release IL-6 by keratinocytes might be supported by extracellular vesicles derived from senescent dermal fibroblasts. The exosomes are also released by melanocytes after exposure to UV radiation [187]. They contain specific miRNAs encoding SASP and possess activities in inducing these cells into premature senescence. Recently, lysophosphatidylcholines have been found as universally elevated in senescent fibroblasts [188]. Furthermore, their capability to elicit a chemokine release in non-senescent fibroblasts was also confirmed. The melanocytes-derived SASP (displayed as elevated RANTES and interferon-gamma inducible-protein-10 (IP-10) and decreased growth-regulated oncogene-α (Gro-α) and vascular endothelial growth factor (VEGF)) promotes telomere dysfunction in a paracrine manner and restricts the proliferation of surrounding cells via the triggering of CXCR3-dependent mitochondrial ROS [47]. In addition, CXCR3 was found to be involved in autocrine signaling, which is important for the establishment of melanocyte senescence. The senescent dermal fibroblasts secrete C-C motif chemokine ligand 2 (CCL2), promoting the recruitment of CCR2+CD14+ monocytes into the skin of older donors after saline, air or varicella zoster virus (VZV) antigen injection [189]. The infiltrating monocytes have an increased expression of cyclooxygenase 2 and can inhibit skin-resident memory T cell proliferation via the production of prostaglandin E2. In addition, the aged fibroblast-derived extracellular matrices had an inhibiting effect on the migration of T cell motility, promoting melanoma metastasis [190].

SASP is not a very unambiguous marker of cellular senescence due to its non-specificity and heterogeneity [22]. However, the presence of matrix metalloproteinases (MMPs), chemokines receptors (such as CXCR2), cytokines (such as IL-6 and IL-8) [6,20] and insulin-like growth factor binding protein 7 (IGFBP7) [175] has been used as a marker for senescent dermal fibroblasts and melanocytes in vitro. In vivo, elevated IL-6 has been detected in nevi melanocytes [42], whereas MMPs are detected in chronologically aged and photoaged skin, and are responsible for the breakdown of the extracellular matrix [191]. In addition, MMP-1 expression was reported to be elevated in fibroblasts in aged human skin in vivo, along with its key regulators, transcription factor AP-1 and α2β1 integrin [192]. Moreover, MMP-1-catalyzed collagen breakdown was also suggested to promote MMP-1 expression through a ROS-dependent manner. Senescent fibroblasts also produce complement factor D, which can negatively influence matrix production and promote the degradation of nearby non-senescent fibroblasts in the dermal layer [193]. IL-1α secretion is increased in keratinocytes derived from the skin of an older chronological age, and might be responsible for increased melanogenesis in melanocytes in aged skin [194]. Furthermore, extrinsic factors, such as cigarette smoking, have been suggested to accelerate skin aging through the elevation of MMPs promoting the degradation of collagen, elastic fibers and proteoglycans [195]. IL-1α and IL-1β play a central role in the induction of the synthesis of both fibroblast-derived IL-6 and collagenase/MMP-1 responsible for the breakdown of dermal interstitial collagen in photoaging caused by UVA irradiation [196]. However, the epidermal keratinocytes are the major cellular source of MMPs, including MMP-1, MMP-3 and MMP-9, which are produced in response to the exposure of human skin to solar UV radiation [160]. Moreover, UVB-induced DNA damage in the keratinocytes was reported to initiate MMP-1 release by fibroblasts [197]. On the other hand, UVB radiation induced the synthesis of SASP-related inflammatory mediators prostaglandin E2 (PGE2), IL-8 and IL-6, and reduced lamin B1 levels in human epidermal keratinocytes [198]. UV irradiation also causes gene mutations in key elements of the TGFβ signaling pathway, including *TGFβRI*, *TGFβRII*, *SMAD2* and *SMAD4*, resulting in the promotion of cancer development [199], as well as photoaging and chronological aging through a reduction in type I procollagen synthesis [200]. Urban dust and diesel exhaust only stimulated the synthesis of IL-8, whereas cigarette smoke extract only stimulated levels of PGE2 in keratinocytes [198]. A combination of topical particulate matter 2.5 (PM_2.5_) and UV exposure induced IL-8 in the 3D skin equivalent model.

The senescent cells can importantly also affect skin-resident immune cells through the promotion of abnormal inflammation interfering with proper adaptive immunity and effective immunosurveillance mechanisms (Figure 3). In particular, raised skin aging-related inflammation can inhibit the response to the challenge with cutaneous antigens, such as VZV antigen [201]. This defect was suggested to be caused, in part, through inhibition by CD4+Foxp3+ regulatory T cells, which can increasingly accumulate in the normal skin of older humans and directly inhibit TNF-α secretion by macrophages [202,203]. Consistently, it can be reversed by the inhibition of inflammatory cytokine production with an oral small-molecule p38 MAPK inhibitor [204]. In addition, as discussed above, an increased expression of PD-1 on CD4+ T cells was also observed in cutaneous aging [170]. Nevertheless, recent data show that the frequency of epidermal CD49a+ CD8+ resident memory T cells was increased in elderly individuals, regardless of ethnicity, and the overall cutaneous T cell density, diversity and protective cytokine production appear to be maintained in aged skin [171].

In the aged epidermis, antigen-presenting cells, namely Langerhans cells (LCs), are less abundant in number (correlating with the age-related decline in granulocyte–macrophage colony-stimulating factor expression [205]). They are also less able to migrate from the epidermis in response to trauma or TNF-α, a key LC mobilization signal, which is attributed to the reduced disposal of local IL-1β [206,207]. The lessened number of LCs in the aged epidermis not only impairs the skin’s ability to regulate immune responses (with likely implications for reduced vaccination efficacy [208]), but can also contribute to the reduced barrier integrity of elderly skin [209], as well as to diminished antimicrobial and tumor cell defense [210].

However, importantly, the persistent cutaneous chronic inflammation levels have been associated with the aging of macrophages [211]. In this regard, skin-resident macrophages display a shift towards pro-inflammatory phenotypes, which promote further tissue inflammation in the skin microenvironment through the secretion of pro-inflammatory cytokines, activation of important inflammatory pathways and increased oxidative stress.

#### 2.1.5. Metabolism Changes

**Figure 4:**
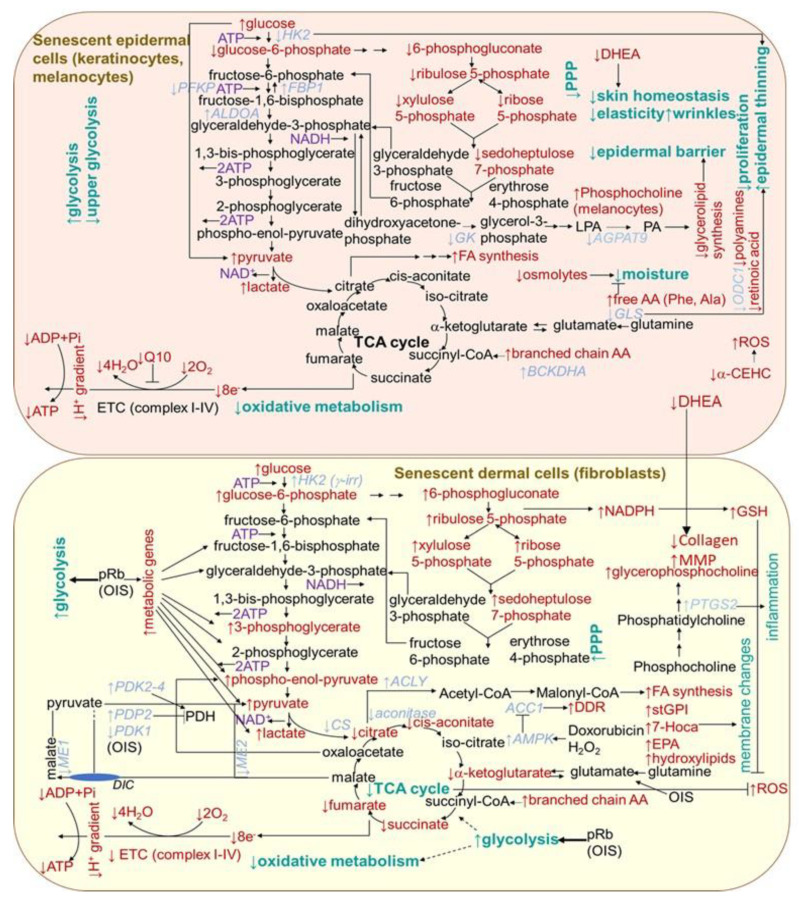
Scheme of aging- and senescence-related changes in metabolic pathways in epidermal and dermal cells. AA, amino acid; ACC1, acetyl-CoA carboxylase; ACLY, ATP citrate lyase; AGPAT9, glycerol-3-phosphate acyltransferase 3; ALDOA, aldolase A; AMPK, 5′ AMP-activated protein kinase; BCKDHA, branched chain keto acid dehydrogenase; α-CEHC, α-carboxyethyl hydroxychroman; CS, citrate synthase; DDR, DNA damage response; DHE, dehydroepiandrosterone; DIC, dicarboxylate carrier; EPA, eicosapentaenoate; ETC, electron transport chain; FA, fatty acid; FBP1, fructose bisphosphatase 1; GLS, glutaminase; GK, glucokinase; HK2, hexokinase 2; 7-Hoca, 7-alpha-hydroxy-3-oxo-4-cholestenoate; LPA, lysophosphatidic acid; ME, malic enzyme; MMP, matrix metalloproteinases; ODC, ornithine decarboxylase 1; OIS, oncogene-induced senescence; PA, phosphatidic acid; PDH, pyruvate dehydrogenase; PDK, pyruvate dehydrogenase kinase; PDP2, pyruvate dehyrogenase phosphatase 2; PFK, phosphofructokinase; PPP, pentose phosphate pathway; pRb, retinoblastoma protein; PTGS2, prostaglandin-endoperoxide synthase 2; stGPI, 1-stearoylglycerophosphoinositol; TCA, tricarboxylic acid cycle.

Metabolic changes in senescent cells are generally documented by an increase in AMP/ATP and ADP/ATP ratios, which is associated with an increase in 5′-adenosine monophosphate-activated protein kinase (AMPK) signaling, leading to the suppression of biosynthetic pathways and activation of catabolic pathways. In support of this, 18 out of 20 genes encoding for mitochondrial complexes I-V were found to be significantly downregulated when comparing between 20- and 70-year-old subjects in the dermal section of the facial cheek photoaged biopsies [212] (Figure 4). The activation of mTOR reduces autophagy, which has an impact on protein homeostasis. Correspondingly, the cells from the dermal tissue of young donors showed a 23% higher level of mitophagy than aged cells from (>75 years old) donors [212]. The p53 has emerged as an essential regulator of metabolic homeostasis, generally, through a suppression glycolysis and an increase in the Krebs cycle, oxidative phosphorylation and fatty acid oxidation [213]. However, senescent fibroblasts in culture are typically more glycolytic than non-senescent cells [214,215], and the oxidative phosphorylation activity seems important in preventing senescence [18]. By contrast, any consistent changes in the expression in mitochondrial-related genes were observed in the epidermal sections from any of the skin biopsy sites [212]. This is in agreement with the dynamic nature of the continually renewing epidermis. Nevertheless, senescence was shown to differentially influence choline metabolism in fibroblasts and melanocytes [216]. Senescent human skin fibroblasts showed elevated levels of glycerol-phosphocholine (GPC). In contrast, melanocytes showed no change in GPC, but a decrease in phosphocholine (PC) levels was detected. In addition, unlike fibroblasts, in senescent melanocytes, the amount of serine, normally needed for their proliferation, decreased. However, in contrast to melanocytes, in fibroblasts, ATP showed lower levels and (−)-inosine showed higher levels in cell senescence. The prevention of the conversion of NAM to NAD^+^ led to premature human primary keratinocyte differentiation and senescence, together with a dramatic drop in glycolysis and cellular ATP levels, while oxidative phosphorylation was modestly affected [217]. However, an increased glycolytic flux and lactate production, as compensation for mitochondrial dysfunction, were reported for keratinocytes from old donors [218] (Figure 4). By contrast, as supported by integrated transcriptome and metabolomic data, the epidermis from the aged donors showed a decreased expression of hexokinase 2 (HK2) (essential for energy generation to support proliferation) correlating with an increased glucose metabolite pool and decreased levels of pentose phosphate pathway metabolites, including sedoheptulose-7-phosphate and pentose-phosphates [219]. In addition, the expression of glycerol-3-phosphate acyltransferase 3 (AGPAT9) and glycerol kinase (GK), linked to glycerolipid biosynthesis was reduced in old skin, suggesting that the epidermal barrier is hampered. The aged epidermis also displayed lower levels of Q10 (essential for optimal mitochondrial function), retinoic acid (necessary for keratinocytes differentiation and proliferation), vitamin E metabolite, 2,5,7,8-tetramethyl-2-(2′-carboxyethyl)-6-hydroxychroman (α-CEHC, providing antioxidant effects in skin), dehydroepiandrosterone (essential for skin homeostasis and mediating collagen synthesis and the regulation of MMP production in the dermis) and organic osmolytes, such as proline betaine (providing moisturizing effect and protection against environmental stresses). Protein synthesis was also shown to be lowered (with a concomitant increase in free amino acids providing an adaptive moisturizing effect), which might be a cause or consequence of the reduced proliferation of keratinocytes. Furthermore, the aged epidermal skin showed decreased transcript levels of ornithine decarboxylase 1 (ODC1), catalyzing the essential step in polyamine synthesis, contributing to a decline in the epidermal cell proliferation.

Further metabolic changes can be indicated by findings from the cellular senescence models established by using diverse human fibroblast cell lines. Extracellular senescence metabolomes (ESMs) from the replicatively senescent human oral fibroblasts and the cells displaying the γ rays-accelerated type of senescence showed an overlap concerning the changes in levels of certain metabolites. In particular, they showed increased levels of citrate, several amino acids including C-glycosyl tryptophan, molecules involved in oxidative stress, a sterol, monohydroxylipids (essential constituents of sphingolipids stabilizing membrane), phospholipids and nucleotide catabolism, as well as diminished levels of dipeptides comprising branched chain amino acids [220]. Moreover, intracellular metabolites of senescent cells indicated an increase in glycolysis, gluconeogenesis, the pentose-phosphate pathway (PPP) and, consistently, a rise in pyruvate dehydrogenase kinase transcripts (Figure 4). In contrast, tricarboxylic acid cycle enzyme transcript levels were unchanged, and their metabolites were depleted. Decreased intracellular citrate levels indicated a decline in mitochondrial metabolism and a reduction in oxidative metabolism. The increased PPP flux was suggested to help to restore redox homeostasis while displaying increased glycolysis in an attempt to avoid further cell damage. Multiple dipeptides were diminished in the senescent cell ESM, probably due to increased catabolism in order to supply carbon skeletons for the tricarboxylic acid (TCA) cycle. Some lipids and their intermediates increased, including 1-stearoylglycerophosphoinositol (stGPI), the sterol 7-alpha-hydroxy-3-oxo-4-cholestenoate (7-Hoca) and eicosapentaenoate (EPA; 20:5 n-3), which is consistent with the increased fatty acid synthesis required for their increase in senescent cell membranes. There were also increased levels of phospholipid catabolites, such as glycerophosphorylcholine (GPC), appearing to correlate with the upregulation of cyclooxygenase 2 gene PTGS2. This is consistent with its association with aging in vivo, since GPC is reduced in the plasma of the long-lived insulin receptor substrate 1 null mouse strain and long-lived dietary-restricted mice [221]. The depletion of thymidine in the medium of senescent cells suggests an increased nucleic acid turnover or altered redox homeostasis in the senescent cells.

The expression of cytosolic malic enzyme 1 (ME1) and mitochondrial malic enzyme 2 (ME2), the key enzymes involved in malate metabolism, exerted a decline in senescent fibroblasts, whereas the overexpression of either enzyme prolonged their replicative lifespan [222]. pRb, being an important regulator of cell cycle arrest during senescence, is also responsible for metabolic changes, as it upregulated a series of glycolytic genes, resulting in increased glycolysis in OIS-induced human lung diploid fibroblasts IMR90 [223]. Consequently, glycolytic stimulation promoted a metabolite flux into the TCA cycle, leading to the OIS-driven activation of mitochondrial oxidative phosphorylation. A decrease in the protein and mRNA levels of acetyl-CoA carboxylase 1 (ACC1) and in lipid synthesis were both found in replicatively senescent human primary fibroblasts IMR90 and in the prematurely senescent cells induced by doxorubicin or hydrogen peroxide [224]. ACC1 decay was also accompanied by the activation of the DNA damage response. By contrast, a number of lipid metabolites appear to be uniquely increased in Ras-induced senescent IMR90 fibroblasts, including a markedly increased number of certain long chain fatty acids [225]. Furthermore, the senescent cells displayed significant changes in lipid metabolism; in particular, a decline in lipid synthesis and a significant increase in fatty acid oxidation. Human fibroblasts cell line TIG3 transformed with BRAF^V600E^, an oncogene-inducing senescence, exert a number of metabolic alterations, comprising augmented oxygen consumption, diminished pyruvate production and an increased production of glutamate [226]. These changes originate from a concurrent restraint of the PDH-inhibitory enzyme pyruvate dehydrogenase kinase 1 (PDK1) and induction of the PDH-activating enzyme pyruvate dehydrogenase phosphatase 2 (PDP2). This results in the enhanced use of pyruvate in the tricarboxylic acid cycle, triggering an increased respiration and redox stress.

#### 2.1.6. Proteostasis Changes

**Figure 5:**
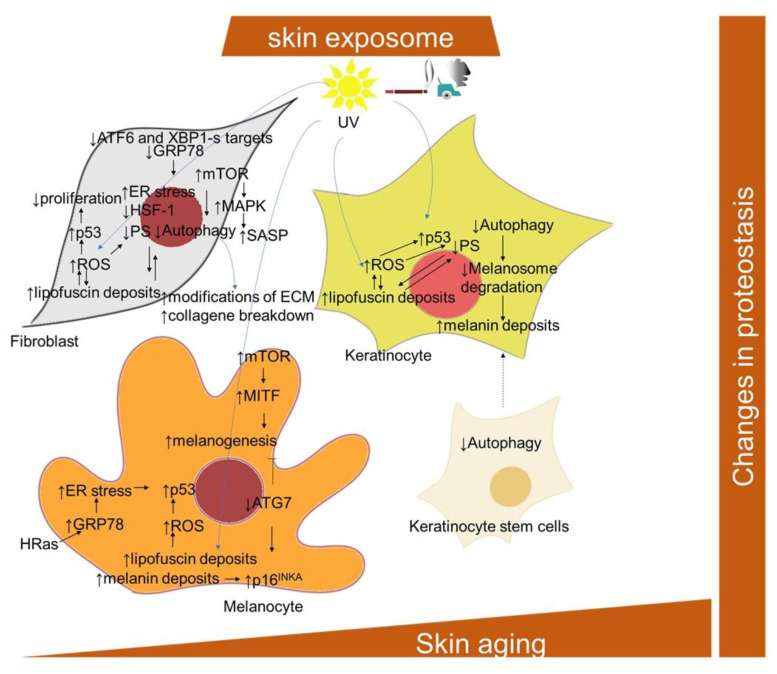
Scheme of the cellular and signaling crosstalk related to age- and senescence-related changes in protein homeostasis in the skin. ATF6, activating transcription factor 6; ATG7, autophagy related 7; GRP78, the 78-kDa glucose-regulated protein; MITF, microphthalmia-associated transcription factor; MAPK, mitogen-activated protein kinase; mTOR, mammalian target of rapamycin; α-MSH, alpha melanocyte stimulating factor; POMC, pro-opiomelanocortin; SDF-1, stromal cell-derived factor-1; XBP1, X-box binding protein 1.

The term proteostasis refers to a balanced and functional cellular proteome, meaning that the response to the protein demands of a cell is optimized for each situation, either by the relocalization of proteins or by tightly regulated cycles of protein synthesis and degradation. During both senescence and aging, there is an increased risk of protein damage, either through oxidation or misfolding, which, in turn, requires either new folding or the degradation of the protein [1]. Proteostasis is maintained by several cellular mechanisms; the main ones are considered to be the ubiquitin–proteasomal system and the autophagy–lysosomal pathway. However, both aging and senescence are associated with significant proteostasis failure, attributed to both autophagy and proteasome dysregulation (Figure 5) [227]. The accumulation of non-functional proteins to form insoluble aggregates has been detected and confirmed in several aging-associated diseases. This indicates a decreased effectiveness of the mechanisms responsible for maintaining proteostasis [228,229,230]. During intrinsic aging and photoaging, markers of protein oxidation were shown to be mainly localized in the dermis (with regard to its low antioxidant levels), while their content in the stratum corneum and in the epidermis remains nearly the same [231]. Accordingly, early- and mid-passage human skin fibroblasts responded to repeated mild heat shock twice a week by an increase in proteasomal activities by 40% to 95% [232]. However, the proteasomal system in late-passage senescent cells appears to be less responsive to the heat shock stimulatory effects. The study of Sabath and colleagues [233] showed that senescent fibroblasts exert an impairment of 160 heat-shock-induced genes, including a number of chaperones, as well as the compromised nuclear translocation and distribution of activated heat shock factor-1 (HSF-1), alternative splicing and coordination of UPR signaling and proteasomal function. Furthermore, ATF6 and XBP1-s target genes, significantly induced in young cells both transcriptionally and translationally, were not induced at all in senescent cells. UV radiation is one of the most relevant factors promoting an increased protein oxidation and proteasome inhibition, which lead to skin aging [234]. In addition, other studies [235,236,237] confirmed that decreased proteasomal activity and proteasomal subunits expression were accompanied by the accumulation of oxidized and ubiquitinated proteins, and with a decreased expression of the proteasomal subunit in chronologically aged fibroblasts and keratinocytes. A proliferation-dependent change in proteasomal transcription and translation, as well as posttranslational changes, such as direct/indirect ROS effects on the proteasome, might explain its age-related activity decline [234]. In addition, fibroblasts treated with proteasome inhibitors exhibit a shortened replicative lifespan and a senescent-like phenotype [238]. Oxidative stress is considered to be one of the main mechanisms activating cellular senescence and intervening skin aging [239,240,241,242,243]. Accordingly, Zglinicki and colleagues [67] showed that artificial lipofuscin, a material naturally made up through oxidation and the crosslinking reaction of proteins in postmitotic cells, can block proliferation in human fibroblasts (Figure 5). The direct inhibitory effect of lipofuscin on proteasome can also contribute to increases in damage accumulation during aging and senescence phenotype development [244]. Comparably, an inverse relationship was found between the SA-β-Gal marker and the proteasome content in serially passaged keratinocytes cultures, as well as in cultures of epidermal cells from aged donors [237]. Moreover, the cells isolated from aged donors displayed increased levels of oxidized and glycated proteins and proteins modified by the lipid peroxidation product 4-hydroxy-2-nonenal. However, the proteasome activity in senescent keratinocytes, measured in permeabilized cell monolayers in situ, decreased relative to the total proteins, but not relative to cell numbers [55]. Moreover, it has been shown that the level of HSP27 is strongly associated with cell senescence. In particular, by using a rat model of photoaged skin, the crucial role of HSP27 in protection from oxidative stress and skin aging after UV irradiation has been suggested [245]. In addition, lipofuscin and melanin deposits generated in UVA-exposed keratinocytes were found to bear properties of an endogenous visible light-sensitive photosensitizer, producing higher levels of singlet oxygen, DNA damage and a wide-range of cellular insults [69].

Three categories of skin cells differentially contributing to the skin aging process with regard to their autophagy inhibition have been suggested by Eckhart and colleagues [246]. In long-lived and mostly quiescent stem cells, the inhibition of autophagy results in their loss and an impaired supply of progeny cells, including keratinocytes. In short-lived differentiating cells, including keratinocytes, sebocytes and sweat gland duct cells, a decline of autophagy is more likely to be inherited from stem cells, and compromise the protective processes against environmental stressors. In long-lived differentiated cells, including melanocytes, fibroblasts, neurons and Merkel cells, autophagy inhibition results in the accumulation of damaged and toxic components and eventual cell death. In this regard, in senescent fibroblasts, high levels of mTOR activity, along with low levels of autophagy-related proteins, ATG5-ATG12, LC3-II/LC3-I ratio, Beclin-1 and p62, may mitigate the effect of autophagy on clearing excessive and damaged proteins and organelles, therefore accelerating the progression of senescence [247,248]. Conversely, rapamycin significantly reduced senescence in UV-treated human dermal fibroblasts, along with the induction of an increase in cell autophagy levels, decrease in the expression of p53, phosphorylation of HSP27, and reduction in genotoxic and oxidative cellular stress [249,250]. The inhibition of autophagy via the depletion of ATG7, ATG12 or lysosomal-associated membrane protein 2 (Lamp2) was also shown to lead to a senescence-like state in two strains of primary human fibroblasts through a ROS- and p53-dependent mechanism [251]. The activation of mTOR reduces autophagy, which has an impact on protein homeostasis [95]. The mTOR signaling has been found to play a role in the regulation of SASP involving MAPK-activated protein kinase 2 and the eukaryotic translation initiation factor 4E-binding protein 1 (4EBP1) in human foreskin fibroblasts BJ and HFFF2 [182]. Moreover, rapamycin, a well-known mTOR inhibitor, induced the downregulation of IL-6 and other cytokine mRNA levels in human foreskin fibroblasts HCA2, but selectively suppressed the translation of the membrane-bound cytokine, IL-1α [181]. The impairment of autophagy in senescent human fibroblasts [252] may also be related to the aberrant deposition of lipofuscin. Deteriorating autophagy in aging dermal fibroblasts was also implicated in another hallmark of skin aging, i.e., modifications of the ECM [253].

ATG7-deleted skin melanocytes developed premature senescence and showed an increased ROS damage and accumulation of ubiquitinated proteins [254]. In addition, the ATG7-deficient melanocytes displayed a senescence-associated secretory phenotype and secreted higher levels of C-X-C motif chemokine ligand -1,-2,-10 and -12 (Cxcl1, Cxcl2, Cxcl10, Cxcl12), which are implicated in the pathogenesis of pigmentary disorders [255]. Furthermore, a deficiency of ATG7-dependent autophagy was shown to downregulate the genes vital for melanogenesis, including MITF (microphthalmia-associated transcription factor) [256]. Nevertheless, increased mTOR signaling during in senescence might be responsible for increased melanogenesis. In this regard, UVB-triggered mTOR signaling, subsequently suppressing autophagy, participates in the upregulation of MITF activity, resulting in melanin production [257]. On the other hand, a senescent phenotype of age-spots-derived keratinocytes could be attributed to intracellular melanin accumulation, since keratinocytes display reduced melanosome degradation via autophagy impairment [258,259].

#### 2.1.7. Endoplasmic Reticulum (ER) Stress

The occurrence of endoplasmic reticulum (ER) stress is particularly relevant in replicatively senescent fibroblasts [260,261], and is compensated for by a cellular response that is called UPR (unfolded protein response), leading to reduced proteosynthesis, an enlarged ER and an export of misfolded proteins [262] (Figure 5). Conceivably, the ER stress can be linked to excessive protein synthesis involved within the SASP program [261,263]. The UPR activation and enlarged ER were also observed in melanocytes undergoing OIS [264]. The central protein in UPR signaling during senescence is probably played by the ER protein of the HSP70 family of chaperones, BiP (binding immunoglobulin protein) [265]. BiP, also known as glucose-regulated protein 78 (GRP78), was found to be downregulated in replicatively senescent human dermal fibroblasts [266]; however, it was increased in the H-Ras V12-induced senescence of melanocytes [264].

#### 2.1.8. Persistent DNA Damage

A senescent phenotype is characterized by a chronic DNA damage response (DDR), and can be identified by the presence of γ-H2AX (phosphorylated form of H2XA visualized as discrete foci using specific fluorescent antibodies), 53BP1 foci [267] and activated ataxia-telangiectasia mutated (ATM) kinase [268]. Double-strand breaks (DSBs) are important activators of DDR, while senescence occurs in the absence of DNA repair. DSBs induce the recruitment of ATM to the site of DNA damage, which, in turn, leads to the phosphorylation of histone H2AX, which facilitates the association of specific DNA repair complexes [268,269]. Histone methylation also contributes to the regulation of this process. For example, histone H3K9 methylation mediates early ATM-mediated DDR signaling; however, it is later removed as part of repair mechanisms. ATM phosphorylates many substrates, including the two essential kinases CHK1 and CHK2, which propagate the phosphorylation cascade and thus DDR signaling [270,271]. The persistent DDR signal induces the phosphorylation of p53, which, in turn, leads to the transcription of many genes [272]. The phosphorylation of p53, as well as the induction of γ-H2AX nuclear foci, are commonly used senescence markers. However, though DDR can induce several DNA-damaging stimuli, it may not result in senescence, and the induction of senescence may not be the result of DDR. Thus, DNA damage itself is not a marker for cellular senescence; however, the occurrence of telomere-associated DNA damage foci could be used as a senescence marker [273,274,275]. By using MRC5 fibroblasts exposed to X-ray irradiation, persistent DNA damage foci were found in X-ray-induced senescent cells, whereas most of the DNA damage foci were detected at telomeres irrespective of telomerase activity [273]. Replicative senescent monkey skin fibroblasts and skin biopsies from aged monkeys showed increased levels of telomere-associated foci, as indicated by the co-localization of γ-H2AX on telomeric DNA [274]. Shortened telomeres have also been detected in skin from aged individuals, in sun-exposed skin and in premalignant skin lesions [276,277]. Other age-related genomic changes have been found in mitochondrial DNA [18], as well as in photoaged human skin [278].

#### 2.1.9. Pigmentation Changes and Skin Cellular Aging

Skin aging is another important process that modifies the pigmentary system of skin, besides UV radiation. The number of melanocytes decreases and the skin color in sun-protected areas lightens with age [258,279]. However, photoaged skin has irregular pigmentation and, frequently, is hyperpigmented. Senile lentigo, also known as age spots, is one of the major signs accompanying wrinkling during the aging of skin. Irregular pigmentation might be attributed to the hyperactivation of melanocytes, altered distribution of pigment and turnover [259]. The accumulation of lipofuscin in senescent cells also contributes to the occurrence of age spots [280].

Melanin is a group of dark pigments synthetized in melanocytes that are able to absorb UV light and, thus, protect the skin from UV radiation. The synthesis of melanin occurs via a biochemical pathway that is named melanogenesis. It takes place in separated lysosome-related organelles (~500 nm in diameter)—melanosomes—in melanocytes, and is transported to keratinocytes; however, the process itself is behind the scope of this review, and it is well described by [281,282,283,284,285]. Mammalian melanin is classified as eumelanin (brown to black color) and pheomelanin (yellow to red color), while human skin contains a mixture of all melanin types. However, eumelanin is the main factor that gives the skin its color [282].

**Figure 6:**
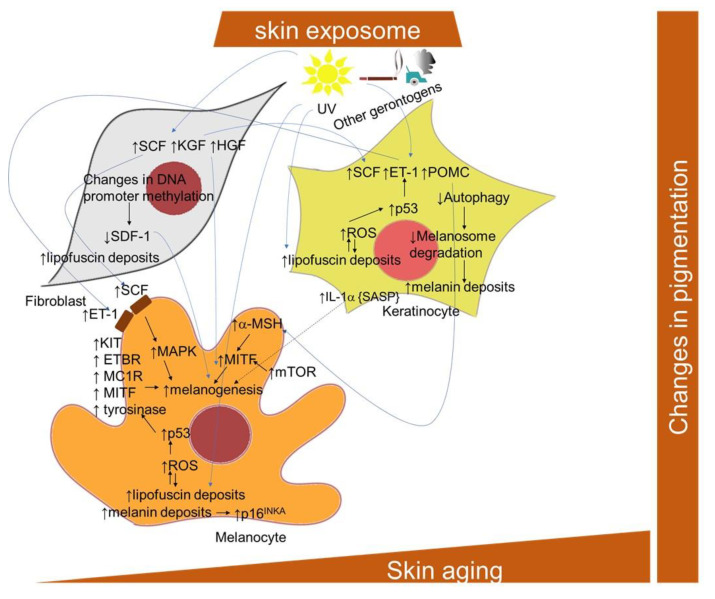
Scheme of the cellular and signaling crosstalk related to age- and senescence-related pigmentation changes in the skin. ET-1, endothelin; ETBR, endothelin–endothelin receptor B; HGF, hepatocyte growth factor; KGF, keratinocyte growth factor; KIT, transmembrane protein with tyrosine kinase activity; MC1R, melanocortin 1 receptor; MITF, microphthalmia-associated transcription factor; a-MSH, alpha melanocyte stimulating factor; POMC, pro-opiomelanocortin; SCF, stem cell factor; SDF-1, stromal cell-derived factor-1.

A marked increase in the eumelanin and total pigment content was found in cultured iridial melanocytes after reaching senescence [286]. However, the levels of pheomelanin remained unaffected. The stimulation of melanin accumulation accelerates melanocyte senescence by a mechanism involving tumor suppressor p16^INK4A^ [287]. Pigmentation is considered an outcome of the interplay between melanocytes and neighboring cells, such as keratinocytes and fibroblasts [288], and both aging and photoaging appear to significantly alter this system (Figure 6). In particular, the development of senescence in keratinocytes and the impaired functioning of autophagy results in a prolonged epidermal retention of melanosomes [259,289]. Interestingly, in a 3D organotypic skin model, the incidence of photo-aged fibroblasts resulted in increased melanogenic gene transcription, increased epidermal melanin and hyperpigmentation [290]. UV irradiation was shown to activate fibroblasts to release melanogenic growth factors, including hepatocyte growth factor (HGF), keratinocyte growth factor (KGF) and stem cell factor (SCF), which act on melanocytes both directly and indirectly through keratinocytes and may contribute to the hyperpigmentation in solar lentigo [291]. In this regard, the essential role of p53 in hyperpigmentation of the skin via the regulation of paracrine signaling mediated by melanogenic factors, including stem cell factor (SCF) and endothelin-1 (ET-1), as well as melanogenic cytokine receptors, was revealed both in keratinocytes and in melanocytes [292]. The role of p53 in the induction of cutaneous pigmentation after UVB irradiation due to the upregulation of pro-opiomelanocortin (POMC) transcript expression in keratinocytes was also demonstrated [293]. Stromal-derived factor 1 (SDF1) deficiency, due to changes in DNA promoter methylation, in senescent fibroblasts, appears to be a potent stimulus for the melanogenic processes that contribute to uneven pigmentation [294]. Furthermore, many premature senescence markers were also found in vitiligo skin, thus confirming that melanocyte functions might be significantly impacted by pathological cross-talk with other cellular components of the skin [295,296].

## 3. Natural Polyphenols against Skin Cellular Aging

The application/administration of natural products, especially botanicals, to improve or eliminate the undesirable signs of aged skin has been used for a very long time [297]. Polyphenols are the largest and most studied group of plant secondary metabolites with known antioxidative properties. They can be categorized as phenolic acids, flavonoids, stilbenes, lignans and other polyphenols with hydroxyl group(s) attached to the carbon atom on the aromatic ring [298]. Today, there is an evidence-based knowledge that the topical or oral intake of some polyphenol-rich plants can prevent or reduce, besides others, undesirable conditions of skin aging.

On the cellular level, several polyphenolic extracts or single compounds have been tested to evaluate their impact on senescence development in cells. In the following, we aimed to summarize the recent in vitro studies related to the anti-senescent mechanisms of natural polyphenols carried out on skin cells.

Several in vitro studies have shown the beneficial effects of polyphenols in both proliferatively senescent skin cells and SIPS models. Treatment with polyphenols can prevent or delay cellular senescence and, thus, can exert beneficial effects on skin aging and age-associated skin diseases. The chronic treatment of pre-senescent neonatal human dermal fibroblasts (NHDF) with 1 μM hydroxytyrosol or 10 μM oleuropein aglycone from extra-virgin olive oil has effectively reduced senescent cell numbers, as demonstrated by evaluating SA-β-Gal-positive cells and p16^INK4A^ protein expression [299].

The increased production of SASP components is one of the most relevant hallmarks of senescence. Essentially, the harmfulness of senescent cells consists in the increased production of SASP factors, such as IL-1, IL-6, IL-8, MMP-1 and MMP-3, which can degrade the ECM. If they are not under control, they can cause chronic inflammation of the tissues, leading to age-related changes and diseases. The anti-inflammatory effect of flavonoids apigenin, quercetin, kaempferol, naringenin and wogonin has been tested on bleomycin-induced senescence in human foreskin fibroblasts [300]. In this study, all flavonoids, except naringenin, significantly inhibited the secretion of SASP markers IL-6, IL-8 and IL-1β. Hydroxytyrosol also showed protective potential against UVA-induced cellular aging in human dermal fibroblasts, as it reduced the expression of inflammatory cytokines IL-1β, IL-6 and IL-8, MMP-1 and -3 gene and SA-β-Gal marker in a dose-dependent manner [301]. HaCaT keratinocyte cultivated in the presence of a bergamot polyphenol fraction (BPF) after UVB treatment resulted in the recovery of cell viability through the modulation of the pro-inflammatory cytokine IL-1β. In addition, treatment with BPF was able to restore the telomere length and telomerase activity in cells that were exposed to UVB radiation [302]. Soybean extract has been investigated for its anti-inflammatory properties in UVB-exposed co-cultures of NHDF and keratinocytes [303]. The treatment of co-cultures with 10 mM genistein, the main compound found in soybeans, slightly decreased the levels of the pro-inflammatory cytokine IL-6 and MAPK signaling through the inhibition of the phosphorylation of p38, extracellular-signal-regulated kinase (ERK) and JNK [303]. Furthermore, the inhibition of the UVB-induced accumulation of the pro-inflammatory intracellular IL-1α (icIL-1α) (by facilitating the removal of damaged cells with pro-inflammatory activity via stimulation of UVB-induced apoptosis) has been shown for rooibos methanolic and aqueous extracts and honeybush aqueous extracts in HaCaT keratinocytes [304]. By contrast, in the same study, the treatment of HaCaT keratinocytes with honeybush methanol extracts after UVB exposure reduced caspase-3 activity, thus preventing apoptotic cell death [304]. These findings confirm that polyphenols can protect the skin from the adverse effects of UVA and UVB radiation, such as hyperpigmentation or the risk of skin cancer, by reducing the pro-inflammatory factors of the SASP.

UVB exposure is a frequently used tool to develop stress-induced premature senescence models in vitro [305]. It has been shown that UVB results in DNA damage or DNA photoproducts in the skin that trigger the signaling pathways associated with the initiation of senescence. The study of Britto and colleagues [306] demonstrated that UVB exposure induces dose-dependent cyclobutane pyrimidine dimers in human dermal fibroblasts, the formation of which, however, has been significantly prevented by apigenin pre- and post-treatment. Likewise, several basic studies have demonstrated the photoprotective effects of polyphenols on the skin. Pomegranate fruit extract (10–40 mg/mL) has been used to treat normal human epidermal keratinocytes for 24 h before UVB exposure, and the dose-dependent inhibition of the UVB-mediated phosphorylation of ERKl/2, JNK1/2 and p38 proteins has been detected [307]. A new derivative of phloretin (50–200 mg/mL) has been used to treat UVB-exposed HaCaT keratinocytes, which resulted in decreased DNA damage and a reduced level of phosphorylated p53 and γ-H2AX. An inhibition of the IL-6 and PGE2 release has also been observed [308]. Furthermore, a decreased number of SA-β-Gal-positive cells associated with an elevated cell viability and relieved G1/G0 cell cycle arrest has been observed in UVB-exposed NHDFs pre-treated with salidroside (1–10 mM) [305]. In the same study, salidroside suppressed the UVB-induced expression of CDK inhibitors p21^CIP/Waf1^ and p16^INK4A^; and it reduced the activity of MMP-1, as well as the production of IL-6 and TNF-α [305].

The increased binding of NF-κB to DNA in the nucleus is one of the most important hallmarks of aging. Indeed, NF-κB is a critical transcription factor involved in the production of SASP and the pathogenesis of many age-related disorders. It has been shown that polyphenols can disrupt the activation of NF-κB and related pathways. Grape seed proanthocyanidins have been reported to inhibit UV-exposure-induced oxidative stress and the activation of MAPK and NF-κB activity in human epidermal keratinocytes [309]. In line with these findings, the interaction of NF-κB and apigenin has been shown to be the crucial mechanism for reducing the secretion of SASP factors [300]. Furthermore, in SKU-1064 skin fibroblasts exposed to UVB and treated with pomegranate extract (5 to 60 mg/L), a protective effect related to a reduced activation of NF-κB, a downregulation of proapoptotic caspase-3 and an increased G0/G1 phase arrest associated with DNA repair has been observed [310].

The free radical theory is one of the most convincing theories in the context of cellular senescence and organismal aging [311,312]. The ROS production, caused by several pathologies, such as mitochondrial damage or endoplasmatic reticulum stress, during the senescence, results in DNA damage and an increased expression of CDK inhibitors, including p21^CIP/Waf1^ or p16^INK4A^. The increased levels of ROS production can be mitigated by certain polyphenols. Glycyrrhizic acid has restored the ROS levels and intracellular Ca^2+^ levels enhanced by UVB exposure in Hs68 foreskin fibroblasts [313]. Moreover, in the same study, glycyrrhizic acid (10 or 25 mM) reduced the phosphorylation of p38, JNK and MAP/ERK kinase (MEK), thus blocking the MAPK signaling pathway. Similarly, decreased IL-6 levels, together with decreased MMP-1 and ROS production, as a consequence of the decreased phosphorylation of AP-1 transcription factor proteins c-Jun and c-Fos, have been observed in UVB-exposed NDHD and HaCaT keratinocytes treated with gallic acid [314]. Furthermore, a suppressed cytoplasmic ROS production accompanied with decreased MMP-1 activity has been detected in piceatannol-treated and UVB-exposed normal human keratinocytes [315]. Furthermore, fisetin has decreased UVB-induced damage by inhibiting ROS production [316]. In this study, the decreased ROS generation was accompanied with the inhibition of the pro-inflammatory TNF-α and hydrogen peroxide-induced senescence in human keratinocytes through the PI3K/AKT/Nrf-2-mediated pathway. The protective effect of brown pine leaf extract (BPLE) and trans-communic acid (TCA) against the effects of UVB irradiation in HaCaT keratinocytes, and also in human reconstructed skin models, has been investigated [317]. HaCaT keratinocytes treated with BPLE and TCA prior to UVB exposure promoted the inhibition of UVB-induced MMP-1 expression and AP-1 transactivation in a dose-dependent manner [317]. These findings suggest that polyphenols are able to modulate the increased ROS levels in senescent cells.

The modulation of pro-inflammatory gene expression, such as the inhibition of cyclooxygenase-2 (COX-2) or inducible nitric oxide synthase (iNOS), has been shown to be one of the major anti-inflammatory mechanisms of polyphenols [318]. In the study of Yoshizaki and colleagues [319], HaCaT keratinocytes were treated with orange peel extract prior to UVB exposure, where orange peel was able to modulate the UVB-induced inflammatory response and suppression of COX-2 expression, and PGE2 production were observed via PPAR-γ activation [319]. Wogonin downregulated COX-2 and iNOS expression in mouse skin fibroblasts treated with TPA, IL-1β and TNF-α [189]. Furthermore, wogonin affected MMP-1 and IL-6 levels in UVB-induced keratinocytes by the inactivation of the MAPK/AP-1 and NF-κB signaling pathways [190]. Comparably to these findings, baicalin showed anti-inflammatory effects and antioxidant properties by modulating MMP-1 and MMP-3 activity in the fibroblasts exposed to UVB [320]. In addition, a decreased SA-β-Gal-positivity, reduced G0/G1 arrest and decreased expression of CDK inhibitors p16^INK4A^ and p21 ^CIP/Waf1^ were shown in baicalin-treated cells.

Furthermore, the elastic properties of UVB-exposed normal human keratinocytes has been restored after pre- and post-treatment with non-toxic concentrations (5 or 10 mM) of delphinidin, as evaluated by atomic force microscopy [321].

**Figure 7:**
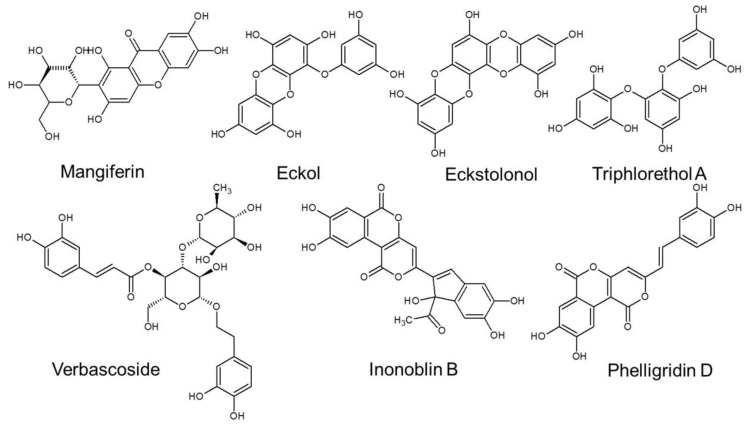
The structures of the selected unique polyphenols exerting protective potential against skin senescence and aging.

The extracts from yerba mate, a tea prepared from the leaves and stems of *Ilex paraguariensis*, obtained after different fermentation times, showed a significant enhancement effect on the cell viability of HaCaT keratinocytes and BJ fibroblasts [322]. The extracts also showed strong inhibitory effects on the activity of lipoxygenase, collagenase and elastase enzymes, as well as the hydration effects on the forearm skin in human volunteers. In addition, the ferments can, through their probiotic activity, support the beneficial microorganisms inhabiting the human skin. Furthermore, mangiferin, a natural polyphenolic compound mainly found in *Mangifera indica*, showed an anti-senescence effect against H_2_O_2_-induced premature senescence in human dermal fibroblast cells (Figure 7). Skin fibroblasts exposed to 10 μM H_2_O_2_ in the presence of 10 μM or 50 μM mangiferin showed a decreased ROS production and stabilized mitochondrial membrane potential, and decreased the number of cell-cycle-arrested cells compared to untreated cells [323].

The extracts from the leaves of *Cleistocalyx nervosum var. paniala*, or Ma Kiang, a perennial tree found growing in scattered locations in the northern provinces of Thailand, showed a promising skin anti-aging potential [324]. In particular, cold methanol extract showed a prominent inhibitory effect on MMP-2, ROS scavenging and lipid peroxidation inhibition, as well as a tyrosinase inhibition effect, along with a low cytotoxicity in human skin fibroblasts. A good photoprotective and antioxidant activity of dried aqueous-methanolic (H_2_O/MeOH) crude extract and ethyl acetate (EtOAc), *n*-butanol (*n*-BuOH), as well as aqueous (H_2_O) fractions of the roots of western Himalayan plant *Potentilla atrosanguinea* (Himalayan cinquefoil), has been proven [325]. In this study, the order of protective efficiencies of the individual extracts was as follows: EtOAc > *n*-BuOH > H_2_O/MeOH > H_2_O. The highest total phenol content was detected in the H_2_O/MeOH extract, showing the highest DPPH radical scavenging, superoxide anion radical scavenging and cupric ion-reducing activity. These findings have suggested the high photoprotective effect applicable in sunscreen preparation.

An extract from tomato (*Lycopersicon esculentum*) stem cells containing both phytochelatin compounds, able to protect the skin from heavy metal toxicity, and polyphenolic antioxidants, has been tested on murine fibroblasts (NIH-3T3) and HaCaT keratinocytes by Tito and colleagues [326]. In their work, it has been demonstrated that a cosmetic product containing this extract can reduce heavy metal-induced toxicity, preserve DNA integrity and decrease collagen degradation by downregulating MMPs. Moreover, it is capable of inducing a new collagen synthesis.

A natural polyphenolic compound, phenylpropanoid glycoside verbascoside (from *Syringa vulgaris* or common lilac), has shown protective effects against UVC-induced damage and pro-inflammatory activation in HaCaT keratinocytes [327] (Figure 7). Verbascoside (100 or 200 μmol/L) added 2 min before irradiation (20 min, 1.8 J/cm^2^) effectively inhibited cytokine-induced proinflammatory molecules and decreased NF-κB and AP-1 DNA binding.

Marine natural products provide a rich source of chemical diversity that can be used to develop novel promising anti-aging skin-care agents. The extracts from three seaweed species of Alariaceae, *Eisenia bicyclis*, *Ecklonia cava* and *Ecklonia stolonifera*, have shown a strong inhibition of both NF-κB and AP-1 reporter activity, which were well correlated with their capabilities to inhibit MMP-1 expression in human dermal fibroblasts [328]. In addition, MMP-1 expression was intensely diminished by treatment with phlorotannins, eckol or dieckol isolated from *E. stolonifera* (Figure 7). In addition, dieckol from *E. cava* showed a tyrosinase inhibition that was relatively higher than that of a commercial tyrosinase inhibitor (kojic acid), and prominent protection of fibroblasts against UV-B radiation-induced DNA damage [329]. In addition, algal phorotannins, including eckstolonol and triphlorethol-A, reduced UV-B-induced reactive oxygen species and nitric oxide levels in zebrafish embryos, protected them against UV-B-induced cell death and significantly reduced hyperpigmentation [330] (Figure 7). The treatment of human diploid fibroblasts WI-38 with phloroglucinol, a basic structural element of phlorotannins, protected cell viability and reduced production of malondialdehyde (MDA), as a marker of lipid oxidation, against premature senescence induced by hydrogen peroxide [331]. Phloroglucinol also alleviated biomarkers of oxidative damage to lipids, proteins and DNA damage in Balb/c mice after UVB radiation [332]. In addition, it decreased the number of mast cells, which are involved in UVB-induced oxidative stress and inflammation, and improved the epidermal and dermal thickness in the dorsal skin of the irradiated mice. These findings indicated that these compounds might represent a promising agent for the prevention and treatment of skin aging and photoaging. A good free radical scavenging ability, antimicrobial activity against *E.coli* and *S. aureus* and effective absorption of the UVB and UVA rays was shown for a polyphenol-rich extract from the seaweed *Sargassum vachellianum*, suggesting its use as a cosmetic ingredient for protecting from photodamage [333].

Furthermore, numerous mushrooms have been used for their beneficial effects of the skin. For instance, the extract from the parasitic mushroom growing on trees, *Inonotus obliquus*, commonly called chaga, has been tested in UV-irradiated skin fibroblasts, keratinocytes or a reconstructed epidermis [334]. In this study, 2% aqueous extract of *Inonotus obliquus* reduced ROS formation in UV-irradiated (UV-A (5 J/cm^2^) + UV-B (100 mJ/cm^2^)) skin cells, which was accompanied with a reduced quantity of pro-inflammatory cytokines and increased DNA repair activity. Compounds responsible for its increased antioxidant activity that were isolated from the *Inonotus obliquus* methanolic extract were inonoblins A, B and C, as well as phelligridin D [335] (Figure 7). Extract of the mycelium of *Tricholoma matsutake*, or pine mushroom, is widely spread in Asian countries. The extract (0.1–100 μg/mL for 72 h) decreased elastase activity and reduced the MMPs levels in human skin fibroblasts, which may suggest its good anti-wrinkle properties [336].

However, it is important to mention that the beneficial effect of polyphenols does not only mean preventing or delaying the outcome of the senescent phenotype, but it also consists of the removal of already senescent cells. The elimination of senescent cells is called senolysis, and many polyphenols, as potential senolytic drugs, also in the context of age-associated deterioration of the skin, are under investigation [337,338,339]. Two established senolytics, a combination of dasatinib plus quercetin (D/Q), significantly reduced the senescent and total cell counts of primary mouse embryonic fibroblasts (MEFs) from *Ercc1^−/−^* mice undergoing premature senescence at passage 5 relative to untreated cultures [144,146]. Quercetin surface-functionalized Fe_3_O_4_ nanoparticles induced a non-apoptotic cell death in oxidative-stress-induced senescent cells through the activation of AMPK [340]. In addition, it also exerted a senostatic effect through decreasing the secreted levels of the IL-8 and IFN-β of the 10 flavonoids tested. Fisetin was the most effective in reducing the fraction of SA-ß-Gal-positive *Ercc1−*/*−*MEFs [341]. In addition, fisetin reduced the senescence in MEFs and IMR90 cells in a dose-dependent manner. Luteolin and curcumin also showed weak senolytic activity in *Ercc1−*/*−*MEFs at a dose where quercetin was ineffective. Nevertheless, the curcumin analog EF24 showed the most potent senolytic activities compared to the other three commonly used curcumin derivatives, HO-3867, 2-HBA and dimethoxycurcumin, in ionizing-radiation-induced senescent WI-38 fibroblasts [342]. EF24 was capable of selective senolysis in various types of senescence through a mechanism that was independent of ROS but dependent on an increase in the proteasome degradation of the Bcl-2 anti-apoptotic protein family proteins, Bcl-xl and Mcl-1. The treatment of senescent and healthy fibroblasts for 48 h with 1% *Rhododendron ferrugineum* leaves extract, which was rich in flavonoids [343], significantly reduced the number of senescent cells while not affecting the number of healthy fibroblasts [344]. This effect was comparable to the effect of the established senolytic drug Navitoclax.

**Figure 8:**
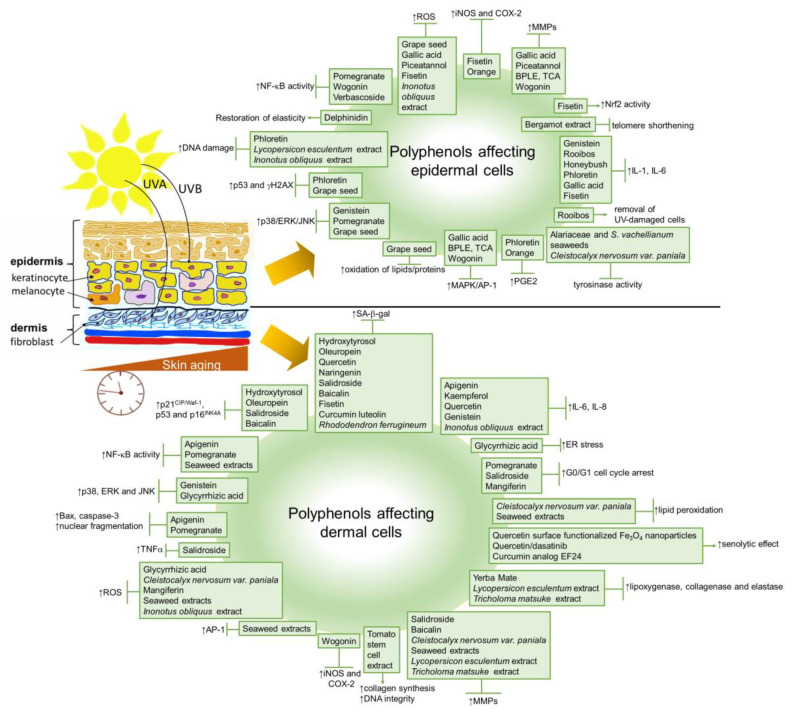
Schematic representation of polyphenols targeting different components/processes of cellular senescence in the skin. AP-1, activator protein 1; BPLE, brown pine leaf extract; COX-2, cyclooxygenase 2; ERK, extracellular-signal-regulated kinase; γ-H2AX, H2AX variant histone; IL-6 and 8, interleukin 6 and 8; JNK, c-Jun N-terminal kinase; MAPK, mitogen-activated protein kinase; MMPs, matrix metalloproteases; NF-κB, nuclear factor kappa B; iNOS, inducible nitric oxide synthase; Nrf2, nuclear factor erythroid 2-related factor 2; SA-β-Gal, senescence associated-beta-galactosidase; PGE2, prostaglandin 2; TNF-α, tumor necrosis factor alpha; TCA, trans-communic acid.

Altogether, polyphenols seem to be effective anti-senescence compounds by targeting critical components of the process of senescence (Figure 8); however, additional studies are required, especially on in vivo models of aged organisms.

Table 1 lists the summary of anti-senescence effects of the above-mentioned polyphenols in in vitro cellular and skin models.

## 4. Polyphenols as Anti-Aging Cosmeceuticals

The aging of the global population puts an increasing demand for not only preventative and therapeutic strategies to combat age-related diseases, but also for cosmetic products containing natural ingredients with active properties that defeat skin aging. In 2011, 63.8% of anti-aging cosmetic products marketed in Europe contained plant-derived preparations, whereas, in 2018, 73.8% of products contained these components [347]. A number of skin care products based on polyphenols or polyphenol-enriched plant extracts, so-termed cosmeceuticals, have been developed with the aim to prevent or delay skin aging [348]. Polyphenols also represent the biologically active constituents of most of the top 10 botanical species, including: *Vitis vinifera* (vine), *Butyrospermum parkii* (shea, or *Vitellaria paradoxa*), *Glycine soja* (soy), *Simmondsia chinensis* (jojoba, or *Buxus chinensis*), *Helianthus annuus* (sunflower), *Theobroma cacao* (cocoa), *Calendula officinalis* (marigold), *Limnanthes alba* (meadowfoam), *Glycyrrhiza glabra* (licorice) and *Acacia decurrens* (black wattle), which are used in anti-aging skin care products [347].

To exert their designated biological activities, topically applied substances aimed to act as anti-aging agents have to be able to be released from the formulation, to reach the skin and, finally, to overcome the stratum corneum barrier and penetrate into the target skin layers, epidermis and dermis.

The release of active substances and further skin permeation depends on the molecule parameters, such as molecular weight and lipophilicity, as well as the vehicle formulation [348]. In the animal skin models, the hydrophobic and a smaller-molecular-weight resveratrol were mostly distributed within the dermis, whereas hydrophilic chlorogenic acid was slightly more distributed in the epidermis [349]. Polyphenols, which are more polar (catechin, resveratrol and curcumin), were mostly concentrated (90%) in the stratum corneum, whereas fewer polar retinols accumulated in the underlying layers of the porcine skin model, and only 10% was retained in the stratum corneum [350].

The suitable vehicle formulation should defeat the polyphenols’ propensity to precipitate in aqueous media due to their poor solubility in water. Emulsions are the most convenient type of formulations designated for topical applications because of their solubilizing capacity for both lipophilic and hydrophilic ingredients [348]. In addition, the incorporation of polyphenols into emulsions can decisively impact their properties, including rheological features, stability and, particularly, the observed decrease in viscosity [351]. Moreover, the stability and activity of polyphenols might also be beneficially modulated by emulsions. For example, the formulation of quercetin to solid dispersions with polyvinylpyrrolidone Kollidon^®^ 25 considerably improved the solubility of quercetin, as well as its antioxidant activity [352]. Furthermore, the components of the emulsions can also influence the release of the polyphenol compounds. For instance, urea and isopropanol hinder the release of rutin from the formulations [353]. However, the presence of propylene glycol 5.0% (*w*/*w*) facilitated the rutin liberation from semisolid systems. The lower oil contents in the emulsions, along with the skin hyperhydration, often promote the release of phenolic substances, as well as higher skin permeation rates [348,354]. Thermodynamically stable microemulsions can also be used as vehicles to boost the skin permeation rates of polyphenols, such as quercetin or chlorogenic acid [355,356]. Other promising skin delivery systems represent nano-emulsions or nanocarriers (involving formulations such as liposomes, niosomes, cubosomes, phytosomes, nanocrystals, polymeric nanoparticles, nanostructured lipid carriers, carbon nanotubes, fullerenes and dendrimers) [357,358] and cosmeto-textiles, as reservoir systems capable of gradual delivery to the skin layers [359,360].

The clinical evidence of the anti-aging effects of plant-based cosmeceuticals is still quite limited. However, some small controlled clinical studies demonstrated the photoprotective or anti-aging effects of topically applied or orally supplemented polyphenols.

The formulations with a content of 2% and 3% green tea extracts (GTEs) showed a prominent protection against skin photo-aging and photoimmunology-related effects in a study involving twenty volunteers exposed to repetitive solar-simulated UV radiation on the upper back at a dosage of 1.5 minimal erythema [361]. The study of Hong and colleagues [362] in forty-two healthy Korean female volunteers, aged 30–59 years, suggested that a treatment of green tea extract with tannase, resulting in an elevation of its constituents gallic acid, epigallocatechin and epicatechin, can increase the rejuvenating effect of GTE on skin. This was demonstrated as a marked or moderate improvement in wrinkles reported by 63.60% of subjects applied with tannase-treated GTE for 8 weeks compared with only 36.30% of reporting women treated with normal GTE. The commercial ginkgo preparation (Flavonoids complex SC^®^) applied for 28 days to the forearm in 20 healthy women aged between 36-and 52-years increased skin moisturization (27.88%) and smoothness (4.32%) and reduced roughness (0.4%) and wrinkles (4.63%). However, a comparable treatment with a formulation containing tea and rooibos (Tealine^®^) showed the best efficacy on wrinkle reduction (9.9%) [363]. In comparison to the tea and rooibos formulation, gingko significantly improved skin moisturization.

Resveratrol, a compound known for its broad range of beneficial biological activities, including its potent antioxidant, anti-inflammatory and modulatory effects on Nrf2 and SirT1, also exerts inhibitory effects on tyrosinase, a key enzyme in the melanin biosynthetic pathway [364]. In a human trial employing 15 healthy volunteers, a stable derivative of resveratrol (resveratrate) exerted protective effects against repetitive solar-simulated UV radiation induced sunburn and suntan [365]. The study involving 20 women showed that the application of a cream containing 0.8% resveratryl triacetate on their face twice daily (morning and evening) for 4 and 8 weeks, in comparison with baseline values before treatment, reduced the total wrinkled area (5.12%, 4.86%), total wrinkle volume (10.53%, 8.41%) and sagging (4.69%, 5.91%), and increased elasticity (2.84%, 3.98%), denseness (15.65%, 20.80%), moisture content (5.83%, 7.37%), lightness (0.79%, 1.07%) and ITA° (a skin color index) (5.43%, 4.95%) [366]. Furthermore, an 8-week application of a new and highly concentrated resveratrol-containing emulsion (Medskin Solutions Dr. Suwelack AG, 2% trans-resveratrol) to 20 subjects resulted in an increase in skin elasticity (+5.3%), and skin density (+10.7%) and a reduction in skin roughness (−6.4%) and skin dispensability (−45.9%) [367]. The facial skin of eight women of ages between 45 and 70 showing clear clinical signs of photoaging presented a remarkable decrease in aging signs, manifested as an increase in luminosity, hydration and elasticity, after the chronic application of trans-resveratrol in combination with beta-cyclodextrin as a carrier twice a day for 1 month [368]. A comparative, randomized and single-blind trial in which 60 female subjects applied twice-daily 1% *Vitis vinifera* shoot extract serum or serum plus cream for four weeks showed a significant improvement in clinical signs of photoaged skin, including skin firmness, radiance, texture, fine lines and wrinkles [369]. The stable water-in-oil emulsion containing 2% Muscat Hamburg grape seed extract topically applied for 8 weeks on the cheek skin of male Pakistani volunteers resulted in a beneficial modulation of facial skin elasticity and content of sebum and melanin [370].

*Calendula officinalis* is a plant that is rich not only in flavonoids but also in terpenoids, carotenoids and volatile oils [371]. A cream containing *C. officinalis* applied to the cheek skin of 21 male volunteers for 8 weeks was found to induce facial skin tightness and hydration, which prevents the damage of skin and also delays the aging process [372]. The clinical study including 12 women aged 30–50 years receiving three concentrations of licorice cream (10%, 20% and 40%) applied on the upper and lower arms twice a day for four weeks showed a decreasing effect of the formulation on the spot pigmentation [373].

Orally supplemented polyphenols or polyphenols-containing dietary supplements can also improve the skin conditions and decelerate the skin aging process. The clinical trial enrolling healthy women of 35–60 years of age showed that a 12-week daily supplementation with two tablets of Imedeen^®^ Time Perfection^®^, a dietary supplement comprising vitamin C, zinc, plant extracts and the proprietary Imedeen marine complex, improved the parameters of photoaged skin, moisturization and skin density [374]. A double-blind placebo-controlled study involving 60 female volunteers aged 40–65 years, documented that a 12-week daily consumption of a beverage with green tea polyphenols comprising 1402 mg total catechins (=1 L of the green tea) can provide protection of skin against harmful UV radiation and can help to improve the overall skin quality of women [375]. The study enrolling 50 healthy males and females aged 35–65 years showed that a 60-day oral supplementation with a nutraceutical supplement containing resveratrol, procyanidin and ellagic acid (present in one daily capsule of Revidox^®^) significantly improved the parameters of skin moisturization and elasticity and diminished the skin roughness, intensity of age spots and wrinkle depth [376]. Interestingly, chronic oral supplementation with Revidox^®^ also improved the antioxidant capacity in *stratum corneum*. The oral supplementation of 30 post-menopausal women with 100 mg/day of an isoflavones-rich, concentrated soy extract for six months increased the epidermal and dermal skin thickness, papillary index, quantity of collagen and elastic fibers and number of dermal vessels in most of the subjects [377].

*Aloe barbadensis* (also known as aloe vera) is a polyphenol-rich plant with proven anti-inflammatory, healing, moisturizing, antibacterial, antifungal, antiviral and anti-aging properties [378,379]. The clinical trial enrolling 30 healthy women over the age of 45 showed that a 90 day intake of a low (120 mL of 1% aloe vera liquid, which is equivalent to 1200 mg of aloe vera gel/day) or high (120 mL of 3% aloe vera liquid, equivalent to 3600 mg of aloe vera gel/day) dose of aloe vera liquid gel (manufacturer: Univera Company, Seoul, Korea) significantly improved facial wrinkles and elasticity, increased type I procollagen and decreased the MMP-1 gene expressions in the photo-protected skin [380].

*Hydrangea serrata*, a plant originating in the East Asia and Korea, but also popular in Europe, has been shown to have positive effects on skin wrinkles, skin hydration, transepidermal water loss and collagen formation in hairless (HR)-1 mice [381]. Furthermore, a randomized, double-blind and placebo-controlled clinical study enrolling 151 healthy male and female volunteers aged 35–60 years receiving a water extract of *Hydrangea serrata* (300 mg, 600 mg or placebo) for 12 weeks confirmed the ability of this polyphenol-rich plant to significantly improve wrinkles, hydration, elasticity, texture and roughness of the skin [382].

The alleviated effects of an apple polyphenol supplement (Applephenon^®^, Asahi Breweries Co. Ltd., Tokyo, Japan) on UV-induced pigmentation has been shown by a randomized, double-blind and placebo-controlled clinical trial enrolling 65 healthy women (age 20–39 years) [383]. In this study, the Applephenon^®^, containing 63.8% procyanidins, 12.4% flavan-3-ols, 10.8% hydroxycinnamic acids and 6.5% phloretin glucosides, was administered as 300 or 600 mg/day once daily for 12 weeks.

*Theobroma cacao*, better known as cocoa, and its products, contain more polyphenolic antioxidants than most food; in particular, flavanols [384]. A randomized, double-blind and placebo-controlled clinical study enrolling 64 Korean women between the ages of 43 and 86 years documented that the daily consumption of the cocoa beverage (samples Barry Callebaut Belgium N.V.) containing 320 mg of cocoa flavanols for 25 weeks remarkably improved skin wrinkles and elasticity in human skin [385]. In this study, the elasticity of the photo-aged skin began to improve after the 12th week and the effects were maintained for the 24th week of the study.

Mangos, particularly Ataulfo mangos, contain a high number of phenolic compounds, especially gallic acid, chlorogenic acid, protocatechuic acid and vanillic acid. A randomized clinical pilot study enrolling healthy postmenopausal women aged 50 to 70 was conducted to assess 16 weeks of either 85 g or 250 g of mango intake. The intake of 85 g of mangos significantly reduced wrinkles in fair-skinned postmenopausal women; however, an intake of 250 g showed the opposite effect. Therefore, further studies are required [386].

The senolytic effect of the organic Alpen rose (*Rhododendron ferrugineum*) extract, confirmed in senescent fibroblasts in vitro (Table 1), has been tested in a double-blind, placebo-controlled clinical study enrolling 44 Caucasian women between 40 and 65 years with redness on their cheeks [344]. Applying a cream with 2% alpine rose extract on the entire face and neck twice daily for 28 days significantly reduced the skin redness and increased its elasticity. Nevertheless, as cellular senescence biomarkers have not been monitored in this study or in any of those reported in this section, the contribution of the anti-senescent efficacy of these preparations to the anti-aging effect on skin remains to be clarified.

## 5. Cellular Aging of Skin and COVID-19 Pandemic

A recent hot topic in the field of research is the investigation of the severe acute respiratory syndrome coronavirus 2 (SARS-CoV-2), which caused the coronavirus disease-19 (COVID-19) pandemic, infected millions of people and is responsible for millions of deaths all over the world [387].

Up to date, it is known that the entry of the coronavirus into target cells primarily depends on the bond of the virus’ spike proteins to the receptors of the host cells. Angiotensin converting enzyme 2 (ACE2) has been identified as an essential receptor of the SARS-CoV-2 [388,389,390]. ACE2-expressing cells are more susceptible to SARS-CoV-2 infection, since the receptor binding domain of the SARS-CoV-2 spike protein has a high affinity to human ACE2 molecules [391]. The expression of the ACE2 protein and RNA have been widely investigated and detected, particularly in the cells of the lung, heart and kidney, as well as those of the skin [390,392,393].

The importance of the renin–angiotensin system, which is responsible, among others, for cell proliferation and differentiation, is detectable also in the skin, where the epidermal stem cells express key mediators of this system, including ACE2 [394]. Recently, the cutaneous involvement in COVID-19 patients has been analyzed and reported in 20.4% as skin rash, widespread urticaria and chickenpox-like vesicles [395]. More recently, through analyzing ACE2 mRNA expression and the ACE2-positive cell composition in skin tissues, a significantly higher expression of ACE2 in keratinocytes and basal cells than in other cell types, such as fibroblasts and melanocytes, has been shown [396]. This may suggest the skin, and in particular, keratinocytes, are a potential target of SARS-CoV-2 infection [397]. In addition, transdermal transmission might signify a potential risk route for SARS-CoV-2 infection.

The cellular aging of the skin can have a significant contribution to this process through the modulation of ACE2 expression, as well as the impairment of skin barrier functions [398]. Recent data show an increase in ACE2 mRNA levels in the late passage of both human fibroblast (BJ) and human bronchial epithelial cells (HBECs) compared to cells at early passages [399]. More importantly, the *ACE2* promoter responds to the activation of the DDR pathways, and telomere dysfunction is a physiological event that is able to engage the DDR pathways modulating the ACE2 levels. Regardless, cellular senescence has been suggested as a potential mediator of COVID-19 severity in elderly patients [400].

Consistently, the study of Bickler and colleagues [401], using a large dataset of genome-wide RNA-seq profiles derived from human dermal fibroblasts, showed that advanced age is associated with an increased expression of genes that encode proteins interacting with SAR2-CoV-2 proteins, including the ACE2 receptor. However, additional studies are needed.

Moreover, the risk factors for the development of severe respiratory illness include pre-existing chronic age-related illnesses, such as diabetes mellitus, chronic lung disease and cardiovascular disease [402]. However, psoriasis, a skin disease with a prevalence that increases with advancing age [403], also showed a significant promoting effect on the expression of ACE2 in skin, which rendered the patients more prone to SARS-CoV-2 infection [404]. However, biological therapy using secukinumab lowered the expression of ACE2 in the skin of psoriasic patients [405]. Furthermore, a higher expression of *ACE2*- and related genes has been observed in the skin samples of patients with atopic dermatitis [406].

The cutaneous infection of SAR-CoV-2 can also be linked to other virus infections in the elderly. For instance, seroepidemiologic surveys demonstrated that 90–97% of adults that are more than 60 years old were seropositive for EBV [407]. EBV was shown to cause robust increases in ACE2 expression in epithelial cells infected with EBV [408]. Furthermore, such an induction of ACE2 expression by EBV enhanced the specific ACE2-dependent entry of SARS-CoV-2 pseudo-typed virions in EBV-infected cells.

Recently, the association between ACE2 expression and different types of cancers has been investigated by many studies, and ACE2 participation in the pathogenesis of the disease has been suggested [409,410,411]. However, the immunohistochemical evaluation of ACE2 presence in healthy and oncologic patients showed ACE2 in the basal cell layer of the normal epidermis and reduced ACE2 reactivity in patients affected by pre-malignant lesions and non-melanoma malignant skin cancers [412]. Furthermore, a positive correlation has been found between ACE2 and the immunotherapy response, considering ACE2 as a potential protective factor with regard to cancer progression [413].

Finally, yet importantly, so-termed virus-induced cellular senescence, which is indistinguishable from other forms of cellular senescence, can also be a consequence of infection by SARS-CoV-2, much like by other viruses [414].

## 6. Summary and Conclusions

Besides triggering prominent visible manifestations, such as wrinkling, sagging, dryness or age spots, skin aging is associated with an onset of age-related skin disorders and diseases, including dermatoses, infections and malignancies [39]. This may be a consequence of the accumulation of senescent cells in the skin tissue. There is growing evidence that cellular senescence may be an essential mechanism that drives organismal aging. In this review, we summarized the actual knowledge about molecular mechanisms of skin cellular senescence and their contribution to the aging of skin. Furthermore, we provided an overview of the polyphenolic substances that are capable of interfering with senescence development in skin cells, as well as the polyphenols-containing preparations that have shown clinical evidence of anti-aging effects. However, data for many of biochemicals mentioned in this paper were derived from in vitro models; thus, in vivo experiments should be carried out to confirm their anti-senescent effect on skin and/or elucidate their contribution to the anti-aging effects observed in vivo. In addition, the molecular mechanisms through which various polyphenols impact the process of senescence should be investigated in more detail in order to identify their specific molecular targets.

Altogether, natural anti-senescence polyphenols have a great potential to be used in the prevention and treatment of the visible signs of premature and chronological aging and age-related disorders of the skin.

## Figures and Tables

**Table 1:** Polyphenols with anti-senescence potential investigated in skin cells in vitro.

Polyphenol	Type of Skin Cells	Assay Conditions	Effect	Reference
Hydroxytyrosol and Oleuropein	neonatal human dermal fibroblasts	1 μM hydroxytyrosol or 10 μM oleuropein	reduced SA-β-Gal-positive cell number and p16^INK4A^ protein expression	[299]
Apigenin	human foreskin fibroblasts	10 or 20 μM for 24 h co-treated with bleomycin	decreased expression of IL-6, IL-8 and IL-1β mRNA; inhibited NF-κB activity	[300]
	NHDF	15 mM 1 h before and after UVB-exposure	downregulated NER expression; inhibited nuclear fragmentation and Bax and caspase-3 expression	[175]
Kaempferol	human foreskin fibroblasts	10 or 20 μM for 24 h co-treated with bleomycin	decreased expression of IL-6, IL-8 and IL-1β mRNA	[300]
Quercetin	human foreskin fibroblasts	10 or 20 μM for 24 h co-treated with bleomycin	decreased expression of IL-6, IL-8 and IL-1β mRNA; reduced SA-β-Gal	[300]
Naringenin	human foreskin fibroblasts	10 or 20 μM for 24 h co-treated with bleomycin	reduced SA-β-Gal	[300]
Bergamot polyphenol fraction	HaCaT	UVB-exposed	modulation of IL-1β; restored telomere length and telomerase activity	[302]
Genistein	NHDF and keratinocytes co-culture	10 mM for 72 h after UVB exposure	inhibited IL-6 production; inhibited phosphorylation of p38, ERK and JNK	[303]
Rooibos methanolic and aqueous extracts	HaCaT	sub-lethal concentrations (0.05–0.55 mg/mL) for 24 h after UVB exposure	inhibited viability and proliferation facilitating the removal of accumulating icIL-1a	[304]
Honeybush aqueous extracts	HaCaT	0.10–0.79 mg/mL for 24 h after UVB exposure	inhibited icIL-1a accumulation; increased caspase-3 activity in damaged cells (with opposing effect found for methanolic extract)	[304]
Pomegranate fruit extract	NHEK	10–40 mg/mL for 24 h before UVB exposure	inhibited phosphorylation of ERK1 and 2, JNK1 and 2 and p38; inhibited phosphorylation of IκBα and IKKα; inhibited translocation of NF-κB/p65	[307]
	SKU-1064 skin fibroblasts	5–60 mg/L for 2 h after UVB exposure	reduced activation of NF-κB; downregulation of caspase-3; increased G0/G1 phase arrest associated with DNA repair	[310]
Phloretin	HaCaT	50–200 mg/mL 12 h after UVB exposure	decreased DNA damage; reduced phosphorylation of p53 and γ-H2AX; inhibited IL-6 and prostaglandin E2	[308]
Salidroside	NHDF	1–10 mM for 24 h before UVB exposure	recovered viability; decreased SA-β-Gal-positive cells; relieved G1/G0 cell cycle arrest; suppressed p21^CIP/Waf1^ and p16^INK4A^ expression; reduced MMP-1 activity; reduced IL-6 and TNF-α production	[305]
Grape seed proanthocyanidins	NHEK	10–50 mM for 3–6 h before UVB exposure	inhibited intracellular release of H_2_O_2;_ inhibited photo-oxidative damage of lipids and proteins; inhibited oxidative DNA damage; inhibited phosphorylation of ERK1 and 2, JNK and p38	[309]
Glycyrrhizic acid	Hs68 foreskin fibroblasts	10–25 mM for 16 h before UVB exposure	reduced ROS levels; restored Ca^2+^ levels; inhibited ER stress; reduced phosphorylation of p38 and JNK	[313]
Gallic acid	NDHF, HaCaT	0.1–10 mM for 24 h after UVB exposure	decreased IL-6; decreased MMP-1 levels; decreased ROS production; suppressed phosphorylation of AP-1	[314]
Piceatannol	NHEK	0–2 mg/mL for 24 h before UVB exposure	suppressed ROS generation; reduced MMP-1 induction	[315]
Fisetin	HaCaT	1–20 mM for 12 h cotreated with H_2_O_2_ (500 mM) or pre-treatment for 6 h before TNF-α stimulation	reduced ROS production; inhibited IL-1β and IL-6 production; decreased iNOS and COX-2 expression; increased Nrf2-mediated HO-1 expression	[316]
Brown pine leaf extract (BPLE) andtrans-communic acid (TCA)	HaCaT, reconstructed human skin models	BPLE (5, 10 μg/mL) and TCA (5, 10 μM) for 1 h before UVB exposure	inhibited MMP-1 expression; suppressed AP-1 expression; inhibited Akt and PI3K phosphorylation	[317]
Orange peel extract	HaCaT	0.1–10 mg/mL prior to UVB exposure	suppressed COX-2 and PGE2 expression; activation of PPAR-γ	[319]
Wogonin	NIH/3T3 mouse skin fibroblasts	TPA, IL-1β and TNF-α and 10–100 mM wogonin for up to 2 h	decreased COX-2 and iNOS expression	[345]
	HaCaT	0.1–10 mM for 72 h after UVB exposure	inhibited MMP-1 and IL-6; blocked MAPK/AP-1 and NF-κB pathways	[346]
Baicalin	human skin samples	6.25–25 mg/mL after UVB exposure	decreased number of SA-β-Gal-positive cells; reduced G0/G1-phase cells; decreased expression of p16^INK4A^, p21^CIP/Waf1^ and p53; decrease in γ-H2AX levels; decreased expression of MMP-1 and MMP-3	[320]
Delphinidin	HaCaT	5 or 10 µM before or after UVB exposure	restored elastic properties	[321]
Extracts from yerba mate	HaCaT, BJ fibroblasts	100–1000 µg/mL extracts	enhanced viability; inhibited activity of lipoxygenase, collagenase and elastase enzymes	[322]
Extracts from leaves of *Cleistocalyx nervosum var. paniala*	human skin fibroblasts mushroom tyrosinase	0.1 mg/ml	inhibition of MMP-2, ROS scavenging, lipid peroxidation inhibition, tyrosinase inhibition effect	[324]
Mangiferin	human dermal fibroblasts	10 μM/50 μM; 2 h followed by addition of H_2_O_2_ (10 μM)	decreased ROS production, stabilized mitochondrial membrane potential and decreased the number of cell cycle arrested cells	[323]
Extracts from three species of seaweeds Alariaceae, *Eisenia bicyclis, Ecklonia cava* and *Ecklonia stolonifera*; eckol, dieckol, eckstolonol, triphlorethol-A and phloroglucinol	human dermal fibroblasts; HeLa cells transfected with the NF-κB or AP-1 luciferase reporter plasmid DNA; mushroom tyrosinase; B16F10 mouse melanoma cells; Zebrafish embryos; male 7-week-old Balb/c mice	10 μg/mL extracts before treatment with TNF-α (10 ng/mL); exposure to UVB (50 mJ/cm^2^) + phlorotannins (0.5–250 μM); zebrafish embryos preincubated with 50 μM phlorotannins for 1 h; phloroglucinol (10 or 50 mg/mL) applied to dorsal skin plus UVB (30 or 60 mJ/cm^2^)	inhibited MMP-1; blocked AP-1 and NF-κB reporter activities; inhibition of tyrosinase, melanogenesis and DNA damage; reduction in ROS, NO, biomarkers of oxidative damage, cell death and hyperpigmentation in vivo; reduction in number of mast cells and increase in the epidermal and dermal thickness	[328,329,330]
Phloroglucinnol	human WI-38 fibroblasts	10, 25, 50, or 100 μg/mL phloroglucinol for 24 h after tratment with 50 μM H_2_O_2_ for 60 min	decrease in MDA in prematurely senescent cells and viability increase	[331]
Polyphenol-rich extract from the seaweed *Sargassum vachellianum*	free radical scavenging, anti-tyrosinase activity and moisture absorption and retention assay	200–1000 μg/mL	potential in scavenging OH radical, and effective absorption of the UVB and UVA rays	[333]
Polyphenol-rich root extracts from *Potentilla atrosanguinea*	determination of total phenol content; free radical scavenging activity	dried aqueous-methanolic (H_2_O/MeOH) crude extract and ethyl acetate (EtOAc), *n*-butanol (*n*-BuOH), as well as aqueous (H_2_O) fractions of roots were evaluated (200 μg/mL)	H_2_O/MeOH crude extract showed highest antioxidant of DPPH radical scavenging, O_2_^.−^ scavenging and Cu^2+^ reducing activity; photoprotective agents in sunscreen preparation; effective natural antioxidant	[325]
Extract from tomato stem cell (*Lycopersicon esculentum)*	murin fibroblasts NIH-3T3; HaCaT	different concentration of the extract for 12 h or 2 h and/or CuSO_4_ for 30 min	reduced heavy metal-induced toxicity, restored DNA integrity under heavy metal stress; decreased collagen degradation and renewed collagen synthesis	[326]
Verbascoside	HaCaT	100 or 200 μmol/L added 2 min before UVC irradiation (20 min, 1.8 J/cm^2^)	decreased AP-1 and NF-κB and decreased level of proinflammatory mediators	[327]
Extract from the parasitic mushroom *Inonotus obliquus*	skin fibroblasts, keratinocytes or reconstructed epidermis	2% aqueous extract added 2 h before UV irradiation (UV-A (5 J/cm^2^) + UV-B (100 mJ/cm^2^)	reduced ROS formation, reduced quantity of pro-inflammatory cytokines and increased DNA repair activity	[334]
Extract of the mycelium of *Tricholoma matsuke*	human skin fibroblasts	0.1–100 μg/mL for 72 h and 24 h treatment in μcombination with TPA	decreased elastase activity, reduced the MMPs level	[336]
Quercetin surface functionalized Fe_3_O_4_ nanoparticles	senescent human foreskin fibroblasts BJ; senescence induced by 100 μM H_2_O_2_ for 2 h	treatment with 5 μg/mL for 24 h	decreased number of stress-induced senescent cells; promoted AMPK activity; reduced IL-8 and IFN-β	[340]
Quercetin/dasatinib	senescent MEFs from *Ercc1−*/*−* mice	48 h treatment dasatinib (250 nM), quercetin (50 μM)	Reduction in senescent and total cell counts	[144,146]
Fisetin	senescent MEFs from *Ercc1−*/*−* mice, IMR-90 fibroblasts	48 h treatment, 1–15 μM	Reduction in the fraction of SA-ß-Gal-positive cells	[341]
Curcumin luteolin	senescent MEFs from *Ercc1−*/*−* mice	48 h treatment, 5 μM	Reduction in the fraction of SA-ß-Gal-positive cells	[341]
Curcumin analog EF24	senescent WI-38 and IMR-90 fibroblasts; senescence induced by replication, oncogene and IR	72 h treatment	Selective killing of senescent cells; EC_50_ = 0.33–1.74 μM; proteasomal degradation of the Bcl-2 anti-apoptotic protein family proteins; independent of ROS	[342]
*Rhododendron ferrugineum* leaves extract	senescent NHDF; senescence induced by 500 µM H_2_O_2_ for 2 h	48 h treatment, 1% extract	Reduction in SA-ß-Gal-positive cells	[344]

AP-1, activator protein 1; COX-2, cyclooxygenase 2; DPPH, 2,2-diphenyl-1-picryl-hydrazyl-hydrate; ER, endoplasmic reticulum; ERK, extracellular-signal-regulated kinase; HaCaT, human immortalized keratinocytes; HO-1, heme-oxygenase 1; IFN-β, interferon beta; IL-1, -6, -8, interleukin 1, 6, 8; iNOS, inducible nitric oxide synthetase; IR, ionization radiation; JNK, c-Jun N-terminal kinase; MAPK, mitogen-activated protein kinase; MDA, malondialdehyde; MEFs, mouse embryonic fibroblasts; MMP-1, matrix metalloproteinase 1; NHDF, normal human dermal fibroblasts; NHEK, normal human epidermal keratinocytes; NF-κB, nuclear factor kappa-light-chain-enhancer of activated B cells; NO, nitric oxide; Nrf2, nuclear factor erythroid 2–related factor 2; PGE2, prostaglandin 2 PI3K, phosphatidylinositol 3-kinase; PPAR-γ, peroxisome proliferator-activated receptor gamma; ROS, reactive oxygen species; SA-β-Gal, senescence associated b-galactosidase.

## Data Availability

Not applicable.
